# The fractional analysis of thermo-elasticity coupled systems with non-linear and singular nature

**DOI:** 10.1038/s41598-024-56891-9

**Published:** 2024-04-26

**Authors:** Abdur Rab, Shahbaz Khan, Hassan Khan, Fairouz Tchier, Samaruddin Jebran, Ferdous Tawfiq, Muhammad Nadeem

**Affiliations:** 1 Department of Mathematics, Abdul Wali khan University, Mardan, Pakistan; 2 Department of Mathematics, Near East University, Mersin 10, Turkey; 3 Department of Mathematics, King Saud university, 11495 Riyadh, Saudi Arabia; 4 Kabul University, Kabul, Afghanistan; 5 School of Mathematics and Statistics, Qujing Normal University, Qujing, China

**Keywords:** Fractional calculus, Caputo operator, Power series, Laplace transform, Laplace residual power series method, Fractional partial differential equation, Mathematics and computing, Physics

## Abstract

It is mentioned that understanding linear and non-linear thermo-elasticity systems is important for understanding temperature, elasticity, stresses, and thermal conductivity. One of the most crucial aspects of the current research is the solution to these systems. The fractional form of several thermo-elastic systems is explored, and elegant solutions are provided. The solutions of fractional and integer thermo-elastic systems are further discussed using tables and diagrams. The closed contact between the LRPSM and exact solutions is displayed in the graphs. Plotting fractional problem solutions demonstrates their convergence towards integer-order problem solutions for suitable modeling. The tables confirm that greater precision is rapidly attained as the terms of the derived series solution increase. The faster convergence and stability of the suggested method support its modification for other fractional non-linear complex systems in nature.

## Introduction

Many scientists use the core concepts and theory of fractional calculus (FC) to investigate memory-related behaviours and dynamical aspects of scientific phenomena. The fundamental reason for the attraction to fractional operators comes from the fact that the usage of fractional differential and integral operators is related to the great application of different models in design, chemical engineering, physical science, and mathematics. It has been rapidly growing and playing a key role in a variety of sectors, assisting in the modelling of innovative problems linked to memory-based fractal-framed repercussions and heredity-related procedures. The primary objective of introducing a fractional order derivative into the system is to investigate the interplay between longer-range, higher degrees of freedom, decreased imprecision because of the uniqueness of the real-world principal parameters, non-local effects that highlight historical and representative future states, maximum information utilisation, and the fractional order systems as particular instances of the conventional order system. By proposing new ideas, many researchers are laying the groundwork for the growth of FC^1–7^.

The solutions of physical and technical importance, fractional ordinary and fractional partial differential equations (FODEs, FPDEs), and integral equations (FIEs)^8^ have gotten a lot of attention. Because most non-linear fractional-order problems do not have exact solutions, to investigate their approximation solutions, analytical and numerical approaches have been suggested and used. On the other hand, many scholars have studied the mathematical characteristics of FPDEs and tried to solve them.

FC is related to real endeavours, and it is broadly used within chaos theory, optics, nanotechnology, human diseases, and other fields^5,9–15^. The analytical and numerical solutions for the above models play an essential part in depicting the aspect of nonlinear issues in related fields of study.

Coupled one-dimensional nonlinear thermo-elasticity coupled systems can be found in a variety of scientific domains, including solid-state and plasma physics. Because of their importance and applications, thermo-elasticity problems have received a lot of attention. A Coupled linear and nonlinear thermo-elasticity system offers a broad field of study for studying the interaction of the mechanical and thermal domains. The study of associated stresses, thermal conductivity, and temperature elasticity is known as thermo-elasticity. Recently, the investigation of these ideas has piqued the interest of numerous scholars working in many fields related to mathematics. Famous scientists, mathematicians, and engineers were influenced by the certainty of irrational physical behaviour, as depicted by elastic deformations obtained by temperature stresses. For such systems, obtaining exact solutions is challenging. Therefore, various analytical and numerical approaches have been developed to solve FPDEs, including the variational iteration method (VIM)^16^, the Adomian decomposition method (ADM)^17^, the q-homotopy analysis transform method (q-HAM)^18^, the fast element-free Galerkin method^19^, the operational matrix method^20^, the fractional natural decomposition scheme^21^, the Fourier transform technique^22^, the Homotopy perturbation method^23^, the operational calculus method^24^, the Laplace-Sumudu transform method^25^, the multistage differential transformation method^26^, and the iterative reproducing kernel method^27^. These techniques have been developed for the approximate solution of FPDEs.

El-Ajou^28^ was the first to employ the Laplace residual power series method (LRPSM) to explore the exact solitary-form solutions of time FPDEs. LRPSM^29,30^ combines the hybrid form of Laplace transform (LT) and RPSM^31–36^. Initially, LT is utilized in LRPSM to convert the given problem into algebraic equations. Subsequently, RPSM is employed to derive the series solution. Ultimately, inverse LT is used to obtain the approximate result. LRPSM demands fewer calculations, less time, and offers greater precision.

LRPSM is an effective and simple method for generating a power series (PS) solution for FPDEs without requiring linearization, discretization, or perturbation. The approach yields a series of algebraic expressions for determining the PS coefficients. Its key advantage lies in relying on simpler and more accurate derivations compared to other integration-based techniques. The method serves as an alternative approach for solving FPDEs^37,38^. In this article, LRPSM is applied to solve the nonlinear systems that arise in thermo-elasticity. The article presents the generalized LRPSM technique, followed by the application of the LRPSM algorithm to several numerical problems. The results and efficiency of the suggested method are demonstrated through tables and graphs. The graphical representation is comprehensive, and the results closely approximate the actual solution for each target problem. The fractional-order LRPSM solutions prove valuable for analyzing the dynamics of the provided problems.

The current paper’s summary is provided here. “Preliminaries” section discussed some necessary definitions and results from FC theory and “LRPS methodology” section the basic technique is presented, certain test models are used to confirm the efficiency of LRPSM in Section “Numerical problems”, the findings are reviewed in “Results and disscusions” section, and the conclusion is provided in “Conclusion” section.

## Preliminaries

The basic definitions and theorems of fractional derivatives in the Caputo sense are covered in this section.

### **Definition 2.1**

The fractional derivative of a function ${\mathcal {P}}({\omega },{\xi })$ of order ${\mu }$ is expressed as in Caputo sense^39^

1 $$\begin{aligned} ^{C}D^{{\mu }}{\mathcal {P}}({\xi })=\frac{1}{\Gamma (m-\zeta )}\int _{0}^{{\xi }}(\xi -\varpi )^{m-\alpha -1}u(\varpi )^{(m)}d\varpi ,\ \ m-1<\zeta \le m, \ \ \xi >\varpi \ge 0. \end{aligned}$$

### **Definition 2.2**

Suppose that ${\mathcal {P}}({\omega },{\xi })$ is continuous piecewise and having ${\mu }$ as exponential order, LT can be explained as^28^:

2 $$\begin{aligned} {\mathcal {P}}({\omega },{s})=\mathcal {L}_{{\xi }}[{\mathcal {P}}({\omega },{\xi })] =\int _{0}^{\infty }e^{-s{\xi }}{\mathcal {P}}({\omega },{\xi })d{\xi }, \ \ s>{\mu }, \end{aligned}$$

where the inverse LT is given as

3 $$\begin{aligned} {\mathcal {P}}({\omega },{\xi })=\mathcal {L}_{{s}}^{-1}[{\mathcal {P}}]({\omega },{s})] =\int _{l-i\infty }^{l+i\infty }e^{s{\xi }}{\mathcal {P}}({\omega },{s})ds,\ \ l=Re(s)>l_{0}, \end{aligned}$$

The properties of the LT and its inverse are summarised in the following Lemma^40^.

### **Lemma 2.3**

Consider the funtions $\varphi ({\omega },{\xi })$ and ${\mathcal {P}}({\omega },{\xi })$, which are continuous piecewise. Then the following properties of LT are held^28^:

1. $$\mathcal {L}_{{\xi }}[\eta \varphi ({\omega },{\xi })+\lambda {\mathcal {P}}({\omega },{\xi })]=\eta \varphi ({\omega },{s})+\lambda {\mathcal {P}}({\omega },{s})$$, $${\omega }\in \textbf{I}$$, $$s>\xi _{1}$$.
2. $$\mathcal {L}_{{\xi }}^{-1}[\eta \varphi ({\omega },{s})+\lambda {\mathcal {P}}({\omega },{s})]=\eta \varphi ({\omega },{\xi })+\lambda {\mathcal {P}}({\omega },{\xi })$$, $${\omega }\in \textbf{I}$$, $${\xi }\ge 0$$.
3. $$\mathcal {L}_{{\xi }}[e^{\rho {\xi }}{\mathcal {P}}({\omega },{\xi })]={\mathcal {P}}({\omega },{s}-\rho )$$. $${\omega }\in \textbf{I}$$, $$s>\rho +\xi _{1}$$.
4. $\lim _{s\rightarrow \infty }s{\mathcal {P}}({\omega },{s})={\mathcal {P}}({\omega },{0})$, ${\omega }\in \textbf{I}$.where $\varphi ({\omega },{s})=\mathcal {L}_{{\xi }}[\varphi ({\omega },{\xi })]$, and ${\mathcal {P}}({\omega },{s})=\mathcal {L}_{{\xi }}[{\mathcal {P}}({\omega },{\xi })]$ and $\eta ,$ $\lambda$ and $\rho$ are arbitrary constants.

### **Lemma 2.4**

Assume that ${\mathcal {P}}({\omega },{\xi })$ is of exponential order $\xi$ and continuous piecewise, and ${\mathcal {P}}({\omega },{s})=\mathcal {L}_{\xi }[{\mathcal {P}}({\omega },{\xi })]$, we have

1. $$\mathcal {L}_{{\xi }}[J_{\xi }^{\zeta }{\mathcal {P}}({\omega },{\xi })]=\frac{{\mathcal {P}}({\omega },{s})}{s^{\zeta }}, \ \ \beta >0.$$
2. $$\mathcal {L}_{{\xi }}[D_{\xi }^{\zeta }{\mathcal {P}}({\omega },{\xi })]=s^{\zeta }{\mathcal {P}}({\omega },{s}) -\sum _{k=0}^{m-1}s^{\zeta -{k}-1}{\mathcal {P}}^{{k}}({\omega },{0}), \ \ m-1<\zeta \le m.$$
3. $$\mathcal {L}_{{\xi }}[D_{\xi }^{n\zeta }{\mathcal {P}}({\omega },{\xi })]=s^{n\zeta }{\mathcal {P}}({\omega },{s}) -\sum _{{k}=0}^{n-1}s^{(n-{k})\zeta -1}D_{\xi }^{{k}\zeta }{\mathcal {P}}({\omega },{0}), \ \ 0<\zeta \le 1.$$

### Proof

The proof can be found in the Refs.^1,2,39^. $\square$

### Theorem 2.5

Consider the piecewise continuous ${\mathcal {P}}({\omega },{\xi })$ is on $\textbf{I}\times [0,\infty )$. Consider that ${\mathcal {P}}({\omega },{s})=\mathcal {L}_{\xi }[{\mathcal {P}}({\omega },{\xi })]$ has fractional power series (FPS) representation^28^:

4 $$\begin{aligned} {\mathcal {P}}({\omega },{s})=\sum _{i=0}^{\infty }\frac{f_{i}({\omega })}{s^{1+i{\zeta }}}, \ \ 0<\zeta \le 1, {\omega }\in \textbf{I}, s>\xi . \end{aligned}$$

Then, $$f_{i}({\omega })=D_{\xi }^{n{\zeta }}{\mathcal {P}}({\omega },{0})$$.

### Remark 2.6

The inverse LT of the Eq. (4) represented as:

5 $$\begin{aligned} {\mathcal {P}}({\omega },{\xi })=\sum _{i=0}^{\infty }\frac{D_{{\xi }}^{\zeta }{\mathcal {P}}({\omega },{0})}{\Gamma (1+i{\zeta })}{\xi }^{i(\zeta )}, \ \ 0<\zeta \le 1, {\xi }\ge 0. \end{aligned}$$

It is equal to the illustration of the fractional order Taylor’s formula in^41^.

The following Theorem explains and establishes the FPS convergence in the 2.5 Theorem.

### Theorem 2.7

Let the function ${\mathcal {P}}({\omega },{\zeta })$ is piecewise continuous on interval $\textrm{I}\times [0,\infty )$ and of exponential order $\varrho$ can be presented as the fractional expansion in Theorem 1. If $|s\mathcal {L}[D_{\xi }^{{{i}}{\zeta }+1}{\mathcal {P}}({\omega },{\zeta })]|\le \mathcal {M}({\theta })$ on $\textrm{I}\times ({\xi },\gamma ]$, where $0<{\zeta }\le 1$, then the remainder $\mathcal {R}_{n}({\theta },s)$ satisfies the below inequality Theorem which satisfy the following^28^:

6 $$\begin{aligned} |R_{i}({\omega },s)|\le \frac{M({\omega })}{S^{1+(i+1){\zeta }}}, \ \ {\omega }\in \textbf{I}, \ \ \xi <s\le \gamma . \end{aligned}$$

## LRPS methodology

In this section, we will discuss the methodology of LRPSM for general form of nonlinear one dimensional thermo-elasticity coupled system

7 $$\begin{aligned} &D_{{\xi }}^{{\zeta +1}}{{\mathcal {P}}}({\omega },{\xi }) -a\left( \frac{\partial {{\mathcal {P}}}}{\partial {\omega }},{\mathcal {R}}\right) \frac{\partial ^{2}}{\partial {\omega }^{2}}{{\mathcal {P}}}({\omega },{\xi }) +b\left( \frac{\partial {{\mathcal {P}}}}{\partial {\omega }},{\mathcal {R}}\right) \frac{\partial {\mathcal {R}}({\omega },{\xi })}{\partial {\omega }}-h({\omega },{\xi })=0,\\&c\left( \frac{\partial {{\mathcal {P}}}}{\partial {\omega }},{\mathcal {R}}\right) D_{{\xi }}^{{\zeta }}{\mathcal {R}}({\omega },{\xi }) +\frac{\partial ^{2}{{\mathcal {P}}}({\omega },{\xi })}{\partial {\xi }\partial {\omega }} -d({\mathcal {R}})\frac{\partial ^{2}}{\partial {\omega }^{2}}{\mathcal {R}}({\omega },{\xi })-m({\omega },{\xi })=0,~0<\zeta \le 1,\ \ \omega \in \Omega , \ \ \xi >0. \end{aligned}$$

with initial conditions (IC’s)

8 $$\begin{aligned} {{\mathcal {P}}}({\omega },0)=f_{0}({\omega }),\ \ {{\mathcal {P}}}_{{\xi }}({\omega },{\xi })=f_{1}({\omega }), \ \ {\mathcal {R}}({\omega },0)=g_{0}({\omega }), \end{aligned}$$

where ${{\mathcal {P}}}$ and ${\mathcal {R}}$ are displacement and temperature difference respectively, $a\left( \frac{\partial {{\mathcal {P}}}}{\partial {\omega }},{\mathcal {R}}\right) , \ c\left( \frac{\partial {{\mathcal {P}}}}{\partial {\omega }},{\mathcal {R}}\right) , \ d({\mathcal {R}}), \ h({\omega },{\xi }), \ m({\omega },{\xi })$ are smooth functions. Now let us assume the following

9 $$\begin{aligned} &a\left( \frac{\partial {{\mathcal {P}}}}{\partial {\omega }},{\mathcal {R}}\right) =c\left( \frac{\partial {{\mathcal {P}}}}{\partial {\omega }},{\mathcal {R}}\right) =d({\mathcal {R}})=1,\ \ b\left( \frac{\partial {{\mathcal {P}}}}{\partial {\omega }},{\mathcal {R}}\right) =\frac{\partial {{\mathcal {P}}}}{\partial {\omega }}{\mathcal {R}}, \end{aligned}$$

Using Eq. (9) in Eq. (7), we get

10 $$\begin{aligned} &D_{{\xi }}^{{\zeta }+1}{{\mathcal {P}}}({\omega },{\xi })-\frac{\partial ^{2}}{\partial {\omega }^{2}}{{\mathcal {P}}} ({\omega },{\xi })+{\mathcal {R}}\frac{\partial {{\mathcal {P}}}({\omega },{\xi })}{\partial {\omega }} \frac{\partial {\mathcal {R}}({\omega },{\xi })}{\partial {\omega }}-h({\omega },{\xi })=0,\\&D_{{\xi }}^{{\zeta }}{\mathcal {R}}({\omega },{\xi })+\frac{\partial ^{2} {{\mathcal {P}}}({\omega },{\xi })}{\partial {\xi }\partial {\omega }}-\frac{\partial ^{2}}{\partial {\omega }^{2}}{\mathcal {R}} ({\omega },{\xi })-m({\omega },{\xi })=0, \ \ 0<\zeta \le 1, \ \ {\xi }>0. \end{aligned}$$

Using LT to Eq. (10) and using IC’s from Eq. (8), we get

11 $$\begin{aligned} {{\mathcal {P}}}({\omega },{s})&-\frac{f_{0}({\omega })}{s}+\frac{f_{1}({\omega })}{s^{2}} -\frac{1}{s^{\zeta +1}}\left[ \frac{\partial ^{2}}{\partial {\omega }^{2}}{{\mathcal {P}}}({\omega },{s})\right] +\frac{1}{s^{\zeta }}\mathcal {L}_{\xi }\left[ \mathcal {L}_{s}^{-1}\left( {\mathcal {R}}({\omega },{s})\right) \frac{\partial }{\partial {\omega }}\mathcal {L}_{s}^{-1}\left( {{\mathcal {P}}}({\omega },{s})\right) \frac{\partial }{\partial {\omega }}\mathcal {L}_{s}^{-1}\left( {\mathcal {R}}({\omega },{s})\right) \right] \\&-\frac{H({\omega },{s})}{s^{\zeta +1}}=0,\\ {\mathcal {R}}({\omega },{s})&-\frac{g_{0}({\omega })}{s} -\frac{1}{s^{\zeta }}\left[ \frac{\partial ^{2}}{\partial {\omega }^{2}}{\mathcal {R}}({\omega },{s})\right] +\frac{1}{s^{\zeta }}\mathcal {L}_{\xi }\left[ \frac{\partial ^{2}}{\partial {\xi }\partial {\omega }}\mathcal {L}_{s}^{-1} \left( {{\mathcal {P}}}({\omega },{s})\right) \right] -\frac{M({\omega },s)}{s^{\zeta }}=0. \end{aligned}$$

Let the approximate solution of Eq. (11) has the following form

12 $$\begin{aligned} {{\mathcal {P}}}({\omega },s)=&\sum _{i=0}^{\infty }\frac{f_{i}}{s^{{\zeta }+{i}+1}},\\ {\mathcal {R}}({\omega },s)=&\sum _{i=0}^{\infty }\frac{g_{i}}{s^{i{\zeta }+1}},\ \ s>0. \end{aligned}$$

The $jth$-truncated term series are

13 $$\begin{aligned} {{\mathcal {P}}}_{{k}}({\omega },s)=&\frac{f_{0}({\omega })}{s}-\frac{f_{1}({\omega })}{s^{2}} +\sum _{i=1}^{{j}}\frac{f_{i}}{s^{{\zeta }+{i}+1}},\\ {\mathcal {R}}_{{k}}({\omega },s)=&\frac{g_{0}({\omega })}{s}+\sum _{i=1}^{{j}}\frac{g_{i}}{s^{i{\zeta }+1}}. \end{aligned}$$

Laplace residual functions (LRFs)^28^ are

14 $$\begin{aligned} \mathcal {L}_{\xi }Res_{{{\mathcal {P}}}}={{\mathcal {P}}}({\omega },{s})&-\frac{f_{0}({\omega })}{s}+\frac{f_{1}({\omega })}{s^{2}}-\frac{1}{s^{\zeta +1}} \left[ \frac{\partial ^{2}}{\partial {\omega }^{2}}{{\mathcal {P}}}({\omega },{s})\right] \\&+\frac{1}{s^{\zeta +1}}\mathcal {L}_{\xi }\left[ \mathcal {L}_{s}^{-1}\left( {\mathcal {R}}({\omega },{s})\right) \frac{\partial }{\partial {\omega }}\mathcal {L}_{s}^{-1}\left( {{\mathcal {P}}}({\omega },{s})\right) \frac{\partial }{\partial {\omega }}\mathcal {L}_{s}^{-1}\left( {\mathcal {R}}({\omega },{s})\right) \right] +\frac{H({\omega },s)}{s^{\zeta +1}},\\ \mathcal {L}_{\xi }Res_{{\mathcal {R}}}={\mathcal {R}}({\omega },{s})&-\frac{g_{0}({\omega })}{s} -\frac{1}{s^{\zeta }}\left[ \frac{\partial ^{2}}{\partial {\omega }^{2}}{\mathcal {R}}({\omega },{s})\right] \\&+\frac{1}{s^{\zeta }}\mathcal {L}_{\xi }\left[ \mathcal {L}_{s}^{-1}\left( {\mathcal {R}}({\omega },{s})\right) \frac{\partial }{\partial {\omega }}\mathcal {L}_{s}^{-1}\left( {{\mathcal {P}}}({\omega },{s})\right) \frac{\partial ^{2}}{\partial {\xi }\partial {\omega }}\mathcal {L}_{s}^{-1}\left( {{\mathcal {P}}}({\omega },{s})\right) \right] +\frac{M({\omega },s)}{s^{\zeta }}. \end{aligned}$$

And the ${j}th$-LRFs as:

15 $$\begin{aligned} \mathcal {L}_{\xi }Res_{{{\mathcal {P}}},{j}}({\omega },s)={{\mathcal {P}}}_{{j}}({\omega },{s})&-\frac{f_{0}({\omega })}{s}+\frac{f_{1}({\omega })}{s^{2}}-\frac{1}{s^{\zeta +1}} \left[ \frac{\partial ^{2}}{\partial {\omega }^{2}}{{\mathcal {P}}}_{{j}}({\omega },{s})\right] \\&+\frac{1}{s^{\zeta +1}}\mathcal {L}_{\xi }\left[ \mathcal {L}_{s}^{-1}\left( {\mathcal {R}}_{{j}}({\omega },{s})\right) \frac{\partial }{\partial {\omega }}\mathcal {L}_{s}^{-1}\left( {{\mathcal {P}}}_{{j}}({\omega },{s})\right) \frac{\partial }{\partial {\omega }}\mathcal {L}_{s}^{-1}\left( {\mathcal {R}}_{{j}}({\omega },{s})\right) \right] -\frac{H({\omega },s)}{s^{\zeta +1}},\\ \mathcal {L}_{\xi }Res_{{\mathcal {R}},{j}}={\mathcal {R}}_{{j}}({\omega },{s})&-\frac{g_{0}({\omega })}{s} -\frac{1}{s^{\zeta }}\left[ \frac{\partial ^{2}}{\partial {\omega }^{2}}{\mathcal {R}}_{{j}}({\omega },{s})\right] \\&+\frac{1}{s^{\zeta }}\mathcal {L}_{\xi }\left[ \frac{\partial ^{2}}{\partial {\xi }\partial {\omega }}\mathcal {L}_{s}^{-1}\left( {{\mathcal {P}}}_{{j}}({\omega },{s})\right) \right] -\frac{M({\omega },s)}{s^{\zeta }}. \end{aligned}$$

The following list includes some key facts regarding the Laplace residual function that are critical to determining the approximation of the solution^28^.

- $\mathcal {L}_{{\xi }}Res({\omega },s)=0$ and $\lim _{{j}\rightarrow \infty }\mathcal {L}_{{\xi }}Res_{{{\mathcal {P}}},{j}}({\omega },s) =\mathcal {L}_{{{\xi }}}Res_{{{\mathcal {P}}}}({\omega },s)$ for each $s>0.$
- $\mathcal {L}_{{\xi }}Res({\omega },s)=0$ and $\lim _{{j}\rightarrow \infty }\mathcal {L}_{{\xi }}Res_{{\mathcal {R}},{j}}({\omega },s) =\mathcal {L}_{{{\xi }}}Res_{{\mathcal {R}}}({\omega },s)$ for each $s>0.$
- $$\lim _{s\rightarrow \infty }s\mathcal {L}_{{{\xi }}}Res_{{{\mathcal {P}}}}({\omega },s) =0\Rightarrow \lim _{s\rightarrow \infty }s\mathcal {L}_{{{\xi }}}Res_{{{\mathcal {P}}},{j}}({\omega },s)=0.$$
- $$\lim _{s\rightarrow \infty }s\mathcal {L}_{{{\xi }}}Res_{{\mathcal {R}}}({\omega },s) =0\Rightarrow \lim _{s\rightarrow \infty }s\mathcal {L}_{{{\xi }}}Res_{{\mathcal {R}},{j}}({\omega },s)=0.$$
- $$\lim _{s\rightarrow \infty }s^{{\zeta }+{j}+1}\mathcal {L}_{{\xi }}Res_{{{\mathcal {P}}},{j}}({\omega },s) =\lim _{s\rightarrow \infty }s^{{\zeta }+{j}+1}\mathcal {L}_{{\xi }}Res_{{\mathcal {R}},{j}}({\omega },s) =0, \ \ 0<{\zeta }\le 1, \ \ {j}=1,2,3,\ldots$$.
- $$\lim _{s\rightarrow \infty }s^{{j}{\zeta }+1}\mathcal {L}_{{\xi }}Res({\omega },s) =\lim _{s\rightarrow \infty }s^{{j}{\zeta }+1}\mathcal {L}_{{{\xi }}}Res_{{\mathcal {R}},{j}}({\omega },s) =0, \ \ 0<{\zeta }\le 1, \ \ {j}=1,2,3,\ldots$$.

To find the coefficients $f_{i}({\omega })$ and $g_{i}({\omega })$, we recursively solve the following system

16 $$\begin{aligned} &\lim _{s\rightarrow \infty }s^{{\zeta }+{j}+1}\mathcal {L}_{{{\xi }}}Res_{{{\mathcal {P}}},{j}}({\omega },s)=0,\ \ {j}=1,2,\ldots ,\\&\lim _{s\rightarrow \infty }s^{{j}{\zeta }+1}\mathcal {L}_{{{\xi }}}Res_{{\mathcal {R}},{j}}({\omega },s)=0,\ \ {j}=1,2,\ldots . \end{aligned}$$

In the last, we apply inverse LT to Eq. (13), to get the ${j}th$ approximate solutions of ${{\mathcal {P}}}_{{j}}({\omega },{\xi })$ and ${\mathcal {R}}_{{j}}({\omega },{\xi })$.

## Numerical problems

### 4.1 Problem

Nonlinear thermo-elasticity coupled system in one-dimensional is given as:^42^

17 $$\begin{aligned} &D_{{\xi }}^{{\zeta +1}}{{\mathcal {P}}}({\omega },{\xi }) -\frac{\partial ^{2}}{\partial {\omega }^{2}}{{\mathcal {P}}}({\omega },{\xi }) +{\mathcal {R}}\frac{\partial {{\mathcal {P}}}({\omega },{\xi })}{\partial {\omega }}\frac{\partial {\mathcal {R}}({\omega },{\xi })}{\partial {\omega }}+e^{-{\omega }+{\xi }}=0,\\&D_{{\xi }}^{{\zeta }}{\mathcal {R}}({\omega },{\xi })+{\mathcal {R}}({\omega },{\xi })\frac{\partial {{\mathcal {P}}}}{\partial {\omega }}\frac{\partial ^{2} {{\mathcal {P}}}({\omega },{\xi })}{\partial {\xi }\partial {\omega }} -\frac{\partial ^{2}}{\partial {\omega }^{2}}{\mathcal {R}}({\omega },{\xi })+e^{{\omega }-{\xi }}=0, \ \ 0<\zeta \le 1, \ \ {\xi }>0. \end{aligned}$$

Subject to the ICs

18 $$\begin{aligned} {{\mathcal {P}}}({\omega },0)=e^{{\omega }},\ \ {{\mathcal {P}}}_{{\xi }}({\omega },{\xi })=-e^{{\omega }}, \ \ {\mathcal {R}}({\omega },0)=e^{-{\omega }}. \end{aligned}$$

Exact solution for the Eq. (17) is

19 $$\begin{aligned} {{\mathcal {P}}}({\omega },{\xi })=&e^{{\omega }-{\xi }},\\ {\mathcal {R}}({\omega },{\xi })=&e^{{\xi }-{\omega }}. \end{aligned}$$

Using LT to Eq. (17) and using IC’s from Eq. (18), we get

20 $$\begin{aligned} {{\mathcal {P}}}({\omega },{s})&-\frac{e^{{\omega }}}{s}+\frac{e^{{\omega }}}{s^{2}} +\frac{1}{s^{\zeta +1}}\mathcal {L}_{\xi }\Bigg [-\frac{\partial ^{2}}{\partial {\omega }^{2}} \mathcal {L}_{s}^{-1}\left( {{\mathcal {P}}}({\omega },{s})\right) +\mathcal {L}_{s}^{-1} \left( {\mathcal {R}}({\omega },{s})\right) \frac{\partial }{\partial {\omega }}\mathcal {L}_{s}^{-1} \left( {{\mathcal {P}}}({\omega },{s})\right) \\&\times \frac{\partial }{\partial {\omega }}\mathcal {L}_{s}^{-1} \left( {\mathcal {R}}({\omega },{s})\right) +e^{-{\omega }+{\xi }}\Bigg ]=0,\\ {\mathcal {R}}({\omega },{s})&-\frac{e^{-{\omega }}}{s} +\frac{1}{s^{\zeta }}\mathcal {L}_{\xi } \Bigg [-\frac{\partial ^{2}}{\partial {\omega }^{2}}\mathcal {L}_{s}^{-1}\left( {\mathcal {R}}({\omega },{s})\right) +\mathcal {L}_{s}^{-1}\left( {\mathcal {R}}({\omega },{s})\right) \frac{\partial }{\partial {\omega }}\mathcal {L}_{s}^{-1}\left( {{\mathcal {P}}}({\omega },{s})\right) \\&\times \frac{\partial ^{2}}{\partial {\xi }\partial {\omega }}\mathcal {L}_{s}^{-1}\left( {{\mathcal {P}}}({\omega },{s})\right) +e^{{\omega }-{\xi }}\Bigg ]=0. \end{aligned}$$

The $jth$-truncated term series are

21 $$\begin{aligned} {{\mathcal {P}}}_{{k}}({\omega },s)=&\frac{e^{\omega }}{s}-\frac{e^{\omega }}{s^{2}} +\sum _{i=1}^{{j}}\frac{f_{i}({\omega })}{s^{{\zeta }+{i}+1}},\\ {\mathcal {R}}_{{k}}({\omega },s)=&\frac{e^{-{\omega }}}{s} +\sum _{i=1}^{{j}}\frac{g_{i}({\omega })}{s^{i{\zeta }+1}}. \end{aligned}$$

And the ${j}th$-LRFs as:

22 $$\begin{aligned} \mathcal {L}_{\xi }Res_{{{\mathcal {P}}},{j}}={{\mathcal {P}}}_{{j}}({\omega },{s})&-\frac{e^{{\omega }}}{s} +\frac{e^{{\omega }}}{s^{2}}+\frac{1}{s^{\zeta +1}}\mathcal {L}_{\xi } \Bigg [-\frac{\partial ^{2}}{\partial {\omega }^{2}}\mathcal {L}_{s}^{-1}\left( {{\mathcal {P}}}_{{j}}({\omega },{s})\right) +\mathcal {L}_{s}^{-1}\left( {\mathcal {R}}_{{j}}({\omega },{s})\right) \frac{\partial }{\partial {\omega }} \mathcal {L}_{s}^{-1}\left( {{\mathcal {P}}}_{{j}}({\omega },{s})\right) \\&\times \frac{\partial }{\partial {\omega }}\mathcal {L}_{s}^{-1}\left( {\mathcal {R}}_{{j}}({\omega },{s})\right) +e^{-{\omega }+{\xi }}\Bigg ],\\ \mathcal {L}_{\xi }Res_{{\mathcal {R}},{j}}={\mathcal {R}}_{{j}}({\omega },{s})&-\frac{e^{-{\omega }}}{s} -\frac{1}{s^{\zeta }}\mathcal {L}_{\xi }\Bigg [-\frac{\partial ^{2}}{\partial {\omega }^{2}}\mathcal {L}_{s}^{-1} \left( {\mathcal {R}}_{{j}}({\omega },{s})\right) +\mathcal {L}_{s}^{-1}\left( {\mathcal {R}}_{{j}}({\omega },{s})\right) \frac{\partial }{\partial {\omega }}\mathcal {L}_{s}^{-1}\left( {{\mathcal {P}}}_{{j}}({\omega },{s})\right) \\&\times \frac{\partial ^{2}}{\partial {\xi }\partial {\omega }}\mathcal {L}_{s}^{-1} \left( {{\mathcal {P}}}_{{j}}({\omega },{s})\right) +e^{{\omega }-{\xi }}\Bigg ]. \end{aligned}$$

Putting the ${j}th$ truncated term series of Eq. (21) into the ${j}th$ truncated Laplace residual function of Eq. (22), multiplying the resulting expression by $s^{j{\zeta }+1}$ and then solve the systems $\lim _{{j}\rightarrow {\infty }}s^{j{\zeta }+1}\mathcal {L}Res_{{{\mathcal {P}}},{j}}({\omega },{s})=0$ and $\lim _{{j}\rightarrow {\infty }}s^{j{\zeta }+1}\mathcal {L}Res_{{\mathcal {R}},{j}}({\omega },{s})=0$ to find the unknown coefficients ${\kappa }_{j}({\omega })$ and ${\varrho }_{j}({\omega })$ for ${j}=1,2,3,\ldots$, the following are the first few terms of the approximate solutions

23 $$\begin{aligned} {\kappa }_{1}({\omega })=&e^{{\omega }},~{\varrho }_{1}({\omega })=e^{{\omega }},\\ {\kappa }_{2}({\omega })=&-e^{{\omega }},~{\varrho }_{2}({\omega })=e^{{\omega }},\\ {\kappa }_{3}({\omega })=&e^{{\omega }},~{\varrho }_{3}({\omega })=e^{{\omega }},\\ {\kappa }_{4}({\omega })=&-e^{{\omega }},~{\varrho }_{4}({\omega })=e^{{\omega }},\\&\vdots , \end{aligned}$$

Substituting ${\kappa }_{j}({\omega })$ and ${\varrho }_{j}({\omega })$ for ${j}=1,2,3,\ldots$ in Eq. (21), we have

24 $$\begin{aligned} {{\mathcal {P}}}({\omega },s)=&\frac{e^{\omega }}{s}-\frac{e^{\omega }}{s^{2}} +\frac{e^{\omega }}{s^{{\zeta }+2}}-\frac{e^{\omega }}{s^{{\zeta }+3}}+\frac{e^{\omega }}{s^{{\zeta }+4}}+\cdots ,\\ {\mathcal {R}}({\omega },s)=&\frac{e^{-{\omega }}}{s}+\frac{e^{-{\omega }}}{s^{{\zeta }+1}} +\frac{e^{-{\omega }}}{s^{2{\zeta }+1}}+\frac{e^{-{\omega }}}{s^{3{\zeta }+1}}+\frac{e^{-{\omega }}}{s^{4{\zeta }+1}}+\cdots . \end{aligned}$$

Utilizing inverse LT on Eq. (24)

25 $$\begin{aligned} {{\mathcal {P}}}({\omega },{\xi })=&e^{\omega }-{\xi }e^{\omega }+\frac{e^{\omega }{\xi }^{{\zeta }+1}}{\Gamma ({\zeta }+2)} -\frac{e^{\omega }{\xi }^{{\zeta }+2}}{\Gamma ({\zeta }+3)}+\frac{e^{\omega }{\xi }^{{\zeta }+3}}{\Gamma ({\zeta }+4)}+\cdots ,\\ {\mathcal {R}}({\omega },{\xi })=&e^{-{\omega }}+\frac{e^{-{\omega }}{\xi }^{\zeta }}{{\Gamma ({\zeta }+1)}} +\frac{e^{-{\omega }}{\xi }^{2{\zeta }}}{\Gamma (2{\zeta }+1)}+\frac{e^{-{\omega }}{\xi }^{3{\zeta }}}{\Gamma (3{\zeta }+1)} +\frac{e^{-{\omega }}{\xi }^{4{\zeta }}}{\Gamma (4{\zeta }+1)}+\cdots . \end{aligned}$$

Putting ${\zeta }=1$ in Eq. (25), we get the exact solution given in Eq. (19) (Figs. 1, 2, 3, 4, 5, 6, 7, 8).

**Figure 1:**
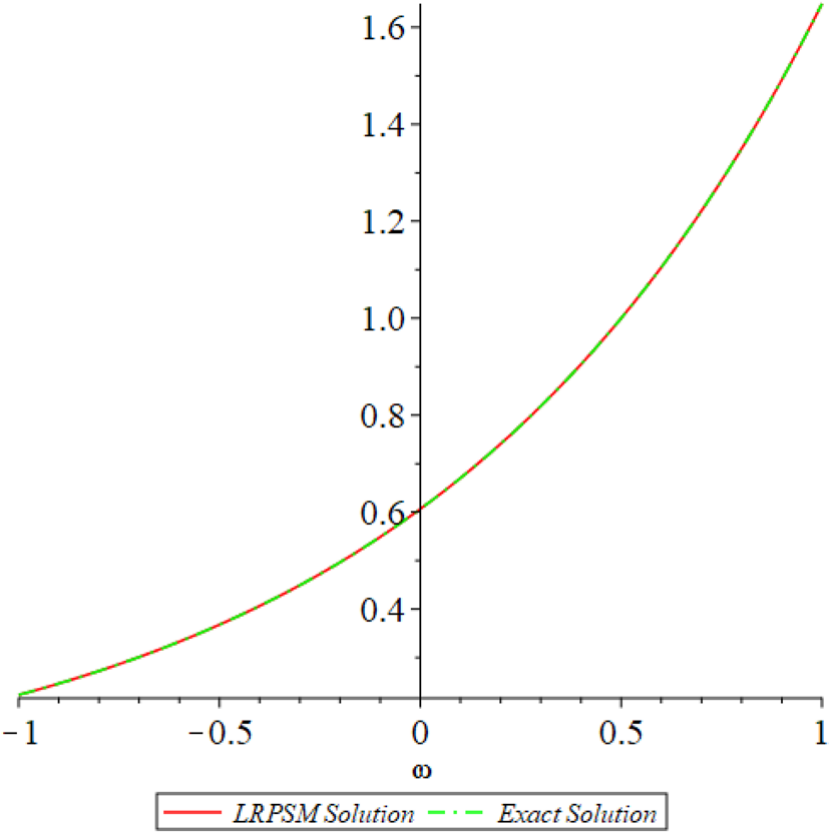
2D graph shows the comparison of LRPSM and exact solutions for ${{\mathcal {P}}}({\omega },{\xi })$ at various fractional order of Example 4.1.

**Figure 2:**
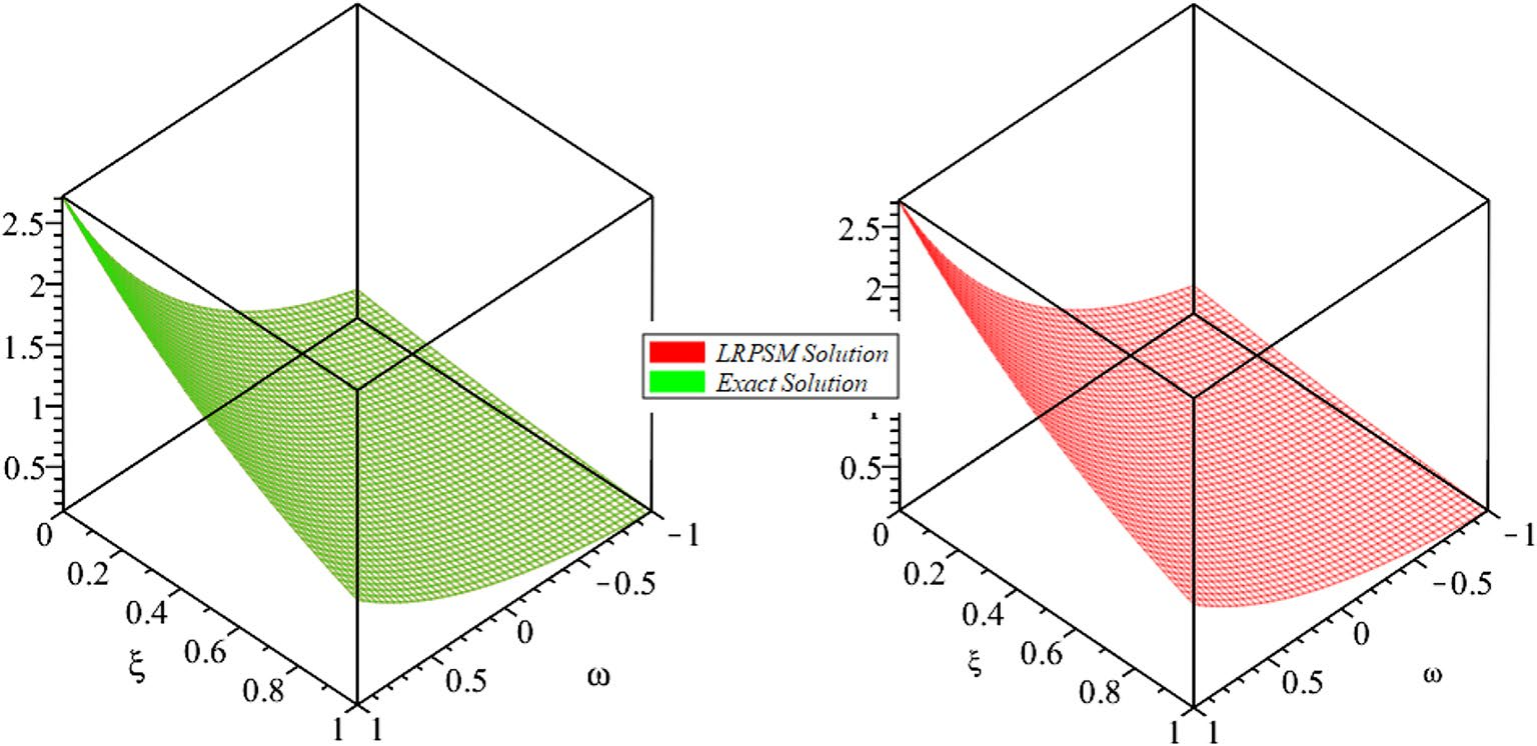
3D surfaces compares the LRSPM and exact solution for ${{\mathcal {P}}}({\omega },{\xi })$ at $\zeta =1$ for problem 4.1.

**Figure 3:**
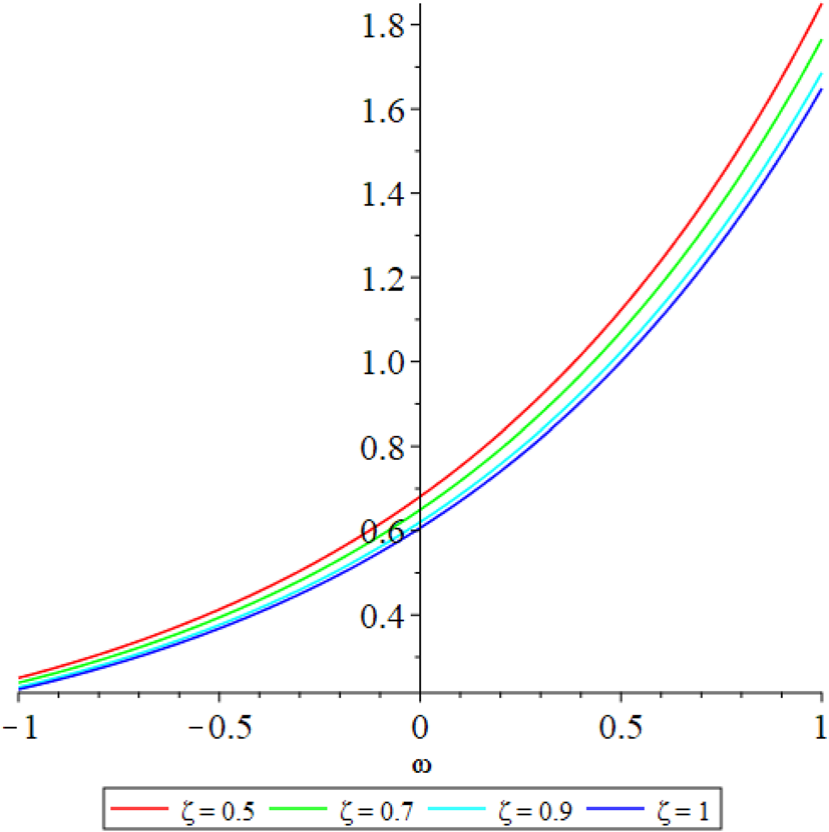
2D graph shows the comparison of ${{\mathcal {P}}}({\omega },{\xi })$ LRPSM solution at various fractional order of Example 4.1.

**Figure 4:**
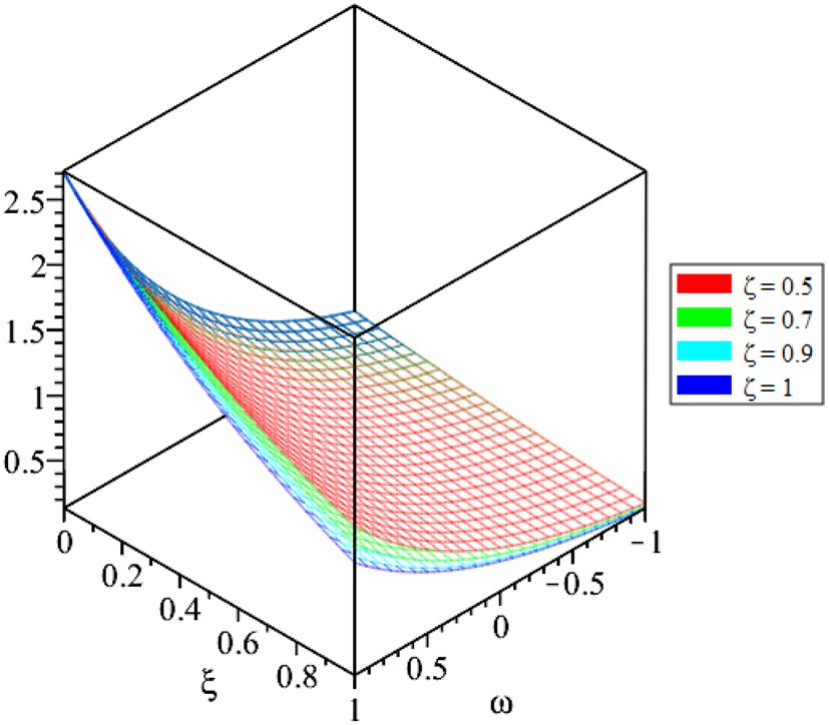
LRPSM solution in 3D surfaces for ${{\mathcal {P}}}({\omega },{\xi })$ at distinct values of $\zeta$ for problem 4.1.

**Figure 5:**
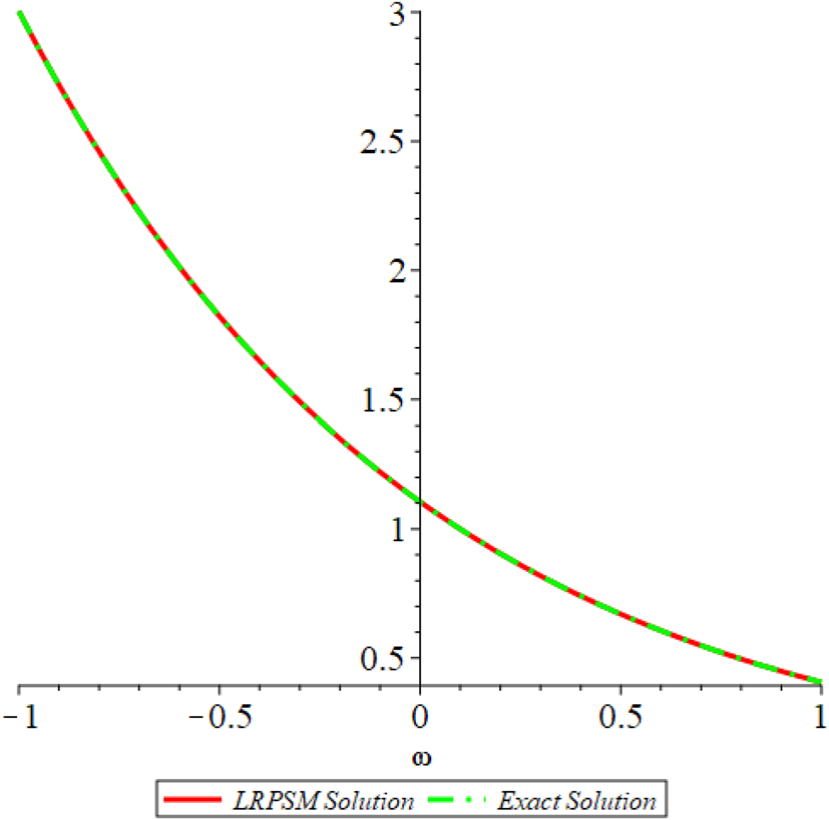
2D graph shows the comparison of LRPSM and exact solutions for ${{\mathcal {R}}}({\omega },{\xi })$ at various fractional order of Example 4.1.

**Figure 6:**
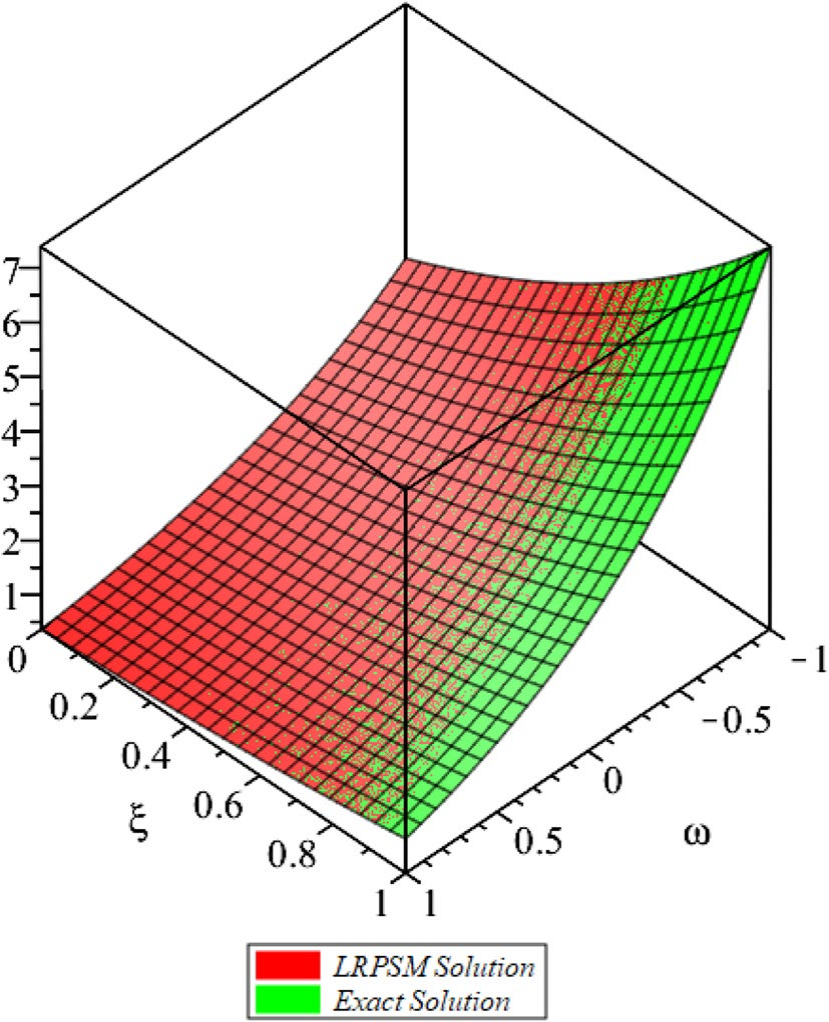
3D surfaces compares the LRSPM and exact solution for ${{\mathcal {R}}}({\omega },{\xi })$ at $\zeta =1$ for problem 4.1.

**Figure 7:**
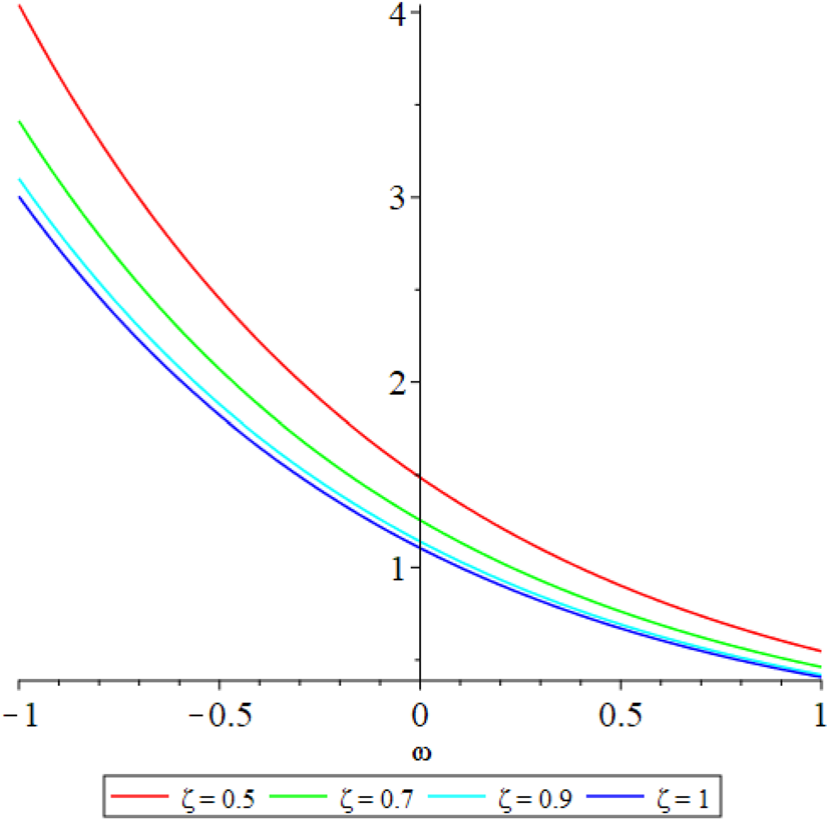
2D graph shows the ${{\mathcal {R}}}({\omega },{\xi })$ LRPSM solution at various fractional order of Example 4.1.

**Figure 8:**
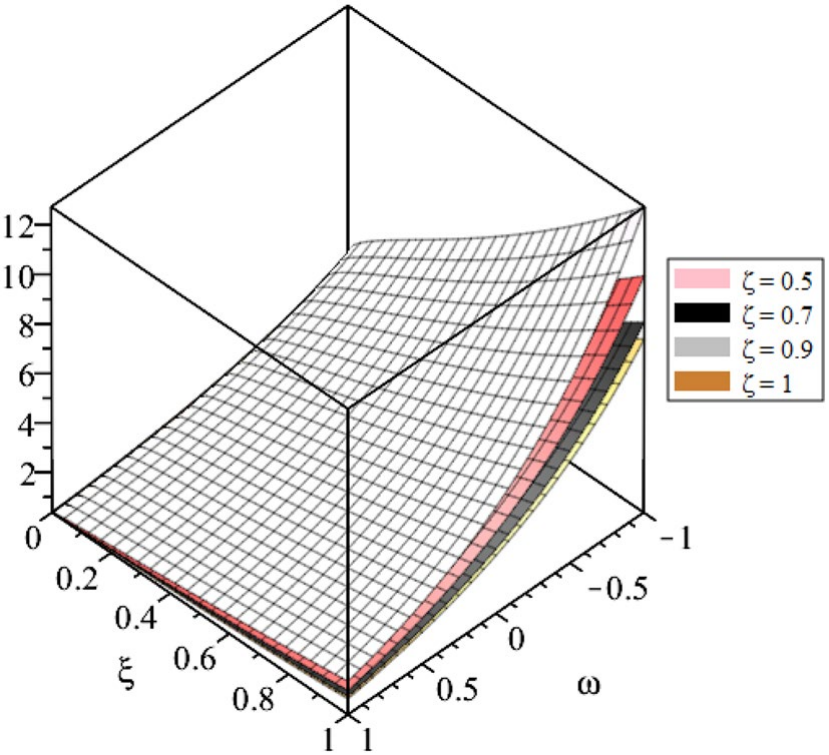
LRPSM solution in 3D surfaces for ${{\mathcal {R}}}({\omega },{\xi })$ at distinct values of $\zeta$ for problem 4.1.

### 4.2 Problem

Nonlinear one-dimensional coupled system thermo-elasticity given by^42^

26 $$\begin{aligned} &D_{{\xi }}^{{\zeta +1}}{{\mathcal {P}}}({\omega },{\xi }) -\frac{\partial }{\partial {\omega }}\left( {\mathcal {R}}({\omega },{\xi }) \frac{\partial }{\partial {\omega }}{{\mathcal {P}}}({\omega },{\xi })\right) +\frac{\partial {\mathcal {R}}({\omega },{\xi })}{\partial {\omega }}-2{\omega }+6{\omega }^{2}+2{\xi }^{2}+2=0,\\&D_{{\xi }}^{{\zeta }}{\mathcal {R}}({\omega },{\xi })-\frac{\partial }{\partial {\omega }}\left( \mathcal {P} ({\omega },{\xi })\frac{\partial }{\partial {\omega }}{\mathcal {R}}({\omega },{\xi })\right) +\frac{\partial ^{2} {{\mathcal {P}}}({\omega },{\xi })}{\partial {\xi }\partial {\omega }}+6{\omega }^{2}-2{\xi }^{2}-2{\xi }=0, \ \ 0<\zeta \le 1, \ \ t>0. \end{aligned}$$

Subject to the ICs

27 $$\begin{aligned} {{\mathcal {P}}}({\omega },0)={\omega }^{2},\ \ {{\mathcal {P}}}_{{\xi }}({\omega },{\xi })=0, \ \ {\mathcal {R}}({\omega },0)={\omega }^{2}. \end{aligned}$$

The exact solution of Eq. (26) is

28 $$\begin{aligned} {{\mathcal {P}}}({\omega },{\xi })=&{\omega }^{2}-{\xi }^{2},\\ {\mathcal {R}}({\omega },{\xi })=&{\omega }^{2}+{\xi }^{2}. \end{aligned}$$

Using LT to Eq. (26) and using IC’s from Eq. (27), we get

29 $$\begin{aligned} {{\mathcal {P}}}({\omega },{s})&-\frac{{\omega }^{2}}{s}+\frac{1}{s^{\zeta +1}}\mathcal {L}_{\xi } \Bigg [-\frac{\partial }{\partial {\omega }}\left( {\mathcal {R}}({\omega },{\xi }) \frac{\partial }{\partial {\omega }}{{\mathcal {P}}}({\omega },{\xi })\right) +\frac{\partial {\mathcal {R}}({\omega },{\xi })}{\partial {\omega }}-2{\omega }+6{\omega }^{2}+2{\xi }^{2}+2\Bigg ]=0,\\ {\mathcal {R}}({\omega },{s})&-\frac{{{\omega }^{2}}}{s}+\frac{1}{s^{\zeta }}\mathcal {L}_{\xi } \Bigg [-\frac{\partial }{\partial {\omega }}\left( \mathcal {P} ({\omega },{\xi })\frac{\partial }{\partial {\omega }}{\mathcal {R}}({\omega },{\xi })\right) +\frac{\partial ^{2} {{\mathcal {P}}}({\omega },{\xi })}{\partial {\xi }\partial {\omega }}+6{\omega }^{2}-2{\xi }^{2}-2{\xi }\Bigg ]=0. \end{aligned}$$

The $jth$-truncated term series are

30 $$\begin{aligned} {{\mathcal {P}}}_{{k}}({\omega },s)=&\frac{{\omega }^{2}}{s}+\sum _{i=1}^{{j}}\frac{f_{i}({\omega })}{s^{{\zeta }+{i}+1}},\\ {\mathcal {R}}_{{k}}({\omega },s)=&\frac{{\omega }^{2}}{s}+\sum _{i=1}^{{j}}\frac{g_{i}({\omega })}{s^{i{\zeta }+1}}. \end{aligned}$$

And the ${j}th$-LRFs as:

31 $$\begin{aligned} \mathcal {L}_{\xi }Res_{{{\mathcal {P}}},{j}}=&{{\mathcal {P}}}_{j}({\omega },{s}) -\frac{{\omega }^{2}}{s}+\frac{1}{s^{\zeta +1}}\mathcal {L}_{\xi }\Bigg [-\frac{\partial }{\partial {\omega }} \left( \mathcal {L}_{s}^{-1}\left( {\mathcal {R}}_{j}({\omega },{\xi })\right) \frac{\partial }{\partial {\omega }}\mathcal {L}_{s}^{-1}\left( {{\mathcal {P}}}_{j}({\omega },{\xi })\right) \right) \\&+\frac{\partial }{\partial {\omega }} \mathcal {L}_{s}^{-1}\left( {\mathcal {R}}_{j}({\omega },{\xi })\right) -2{\omega }+6{\omega }^{2}+2{\xi }^{2}+2\Bigg ],\\ \mathcal {L}_{\xi }Res_{{\mathcal {R}},{j}}=&{\mathcal {R}}_{j}({\omega },{s}) -\frac{{{\omega }^{2}}}{s}+\frac{1}{s^{\zeta }}\mathcal {L}_{\xi }\Bigg [-\frac{\partial }{\partial {\omega }} \left( \mathcal {L}_{s}^{-1}\left( {{\mathcal {P}}}_{j} ({\omega },{\xi })\right) \frac{\partial }{\partial {\omega }}\mathcal {L}_{s}^{-1}\left( {\mathcal {R}}_{j}({\omega },{\xi })\right) \right) \\&+\frac{\partial ^{2}}{\partial {\xi }\partial {\omega }} \mathcal {L}_{s}^{-1}\left( {{\mathcal {P}}}_{j}({\omega },{\xi })\right) +6{\omega }^{2}-2{\xi }^{2}-2{\xi }\Bigg ]. \end{aligned}$$

Putting the ${j}th$ truncated term series of Eq. (39) into the ${j}th$ truncated Laplace residual function of Eq. (40), multiplying the resulting expression by $s^{j{\zeta }+1}$ and then solve the systems $\lim _{{j}\rightarrow {\infty }}s^{j{\zeta }+1}\mathcal {L}Res_{{{\mathcal {P}}},{j}}({\omega },{s})=0$ and $\lim _{{j}\rightarrow {\infty }}s^{j{\zeta }+1}\mathcal {L}Res_{{\mathcal {R}},{j}}({\omega },{s})=0$ to find the unknown coefficients ${\kappa }_{j}({\omega })$ and ${\varrho }_{j}({\omega })$ for ${j}=1,2,3,\ldots$, the following are the first few terms of the approximate solutions

32 $$\begin{aligned} {\kappa }_{1}({\omega })=&-2,\\ {\varrho }_{1}({\omega })=&0,\\ {\kappa }_{2}({\omega })=&2,\\ {\varrho }_{2}({\omega })=&0,\\ {\kappa }_{3}({\omega })=&0,\\ {\varrho }_{3}({\omega })=&0,\\&\vdots , \end{aligned}$$

Substituting ${\kappa }_{j}({\omega })$ and ${\varrho }_{j}({\omega })$ for ${j}=1,2,3,\ldots$ in Eq. (39), we have

33 $$\begin{aligned} {{\mathcal {P}}}({\omega },s)=&\frac{{\omega }^{2}}{s}+\frac{-2}{s^{{\zeta }+2}}+0+0+\cdots ,\\ {\mathcal {R}}({\omega },s)=&\frac{{\omega }^{2}}{s}+\frac{2}{s^{{\zeta }+1}}+0+0+\cdots . \end{aligned}$$

Utilizing inverse LT on Eq. (42), we get the approximate solution as

34 $$\begin{aligned} {{\mathcal {P}}}({\omega },{\xi })=&{\omega }^{2}-\frac{2{\xi }^{{\zeta }+1}}{\Gamma ({\zeta }+2)},\\ {\mathcal {R}}({\omega },{\xi })=&{\omega }^{2}+\frac{2{\xi }^{\zeta }}{\Gamma ({\zeta }+1)}. \end{aligned}$$

Putting $\zeta =1$, we get the exact solution which is given in Eq. (26).

### 4.3 Problem

Consider the singular one-dimensional linear thermo-elasticity coupled system^42^

35 $$\begin{aligned} &D_{{\xi }}^{{\zeta +1}}{{\mathcal {P}}}({\omega },{\xi })-\frac{1}{{\omega }^{2}} \left( {\omega }^{2}\frac{\partial }{\partial {\omega }}{{\mathcal {P}}}({\omega },{\xi })\right) +{\omega }\frac{\partial {\mathcal {R}}({\omega },{\xi })}{\partial {\omega }}-2{\omega }^{2}{\xi }-6-{\xi }=0,\\&D_{{\xi }}^{{\zeta }}{\mathcal {R}}({\omega },{\xi })-\frac{1}{{\omega }^{2}} \left( {\omega }^{2}\frac{\partial }{\partial {\omega }}{\mathcal {R}}({\omega },{\xi })\right) +{\omega }\frac{\partial ^{2} {{\mathcal {P}}}({\omega },{\xi })}{\partial {\xi }\partial {\omega }}-3{\omega }^{2}+6{\xi }=0, \ \ 0<\zeta \le 1, \ \ t>0. \end{aligned}$$

Subject to the ICs

36 $$\begin{aligned} {{\mathcal {P}}}({\omega },0)={\omega }^{2},\ \ {{\mathcal {P}}}_{{\xi }}({\omega },{0})={\omega }^{2}, \ \ {\mathcal {R}}({\omega },0)=0. \end{aligned}$$

Eq. (35) exact solution is as follows:

37 $$\begin{aligned} {{\mathcal {P}}}({\omega },{\xi })=&{\omega }^{2}+{\omega }^{2}{\xi },\\ {\mathcal {R}}({\omega },{\xi })=&{\omega }^{2}{\xi }^{2}. \end{aligned}$$

Using LT to Eq. (35) and using IC’s from Eq. (36), we get

38 $$\begin{aligned} {{\mathcal {P}}}({\omega },{s})&-\frac{{\omega }^{2}}{s} -\frac{{\omega }^{2}}{{s}^{2}}+\frac{1}{s^{\zeta +1}}\mathcal {L}_{\xi }\Bigg [-\frac{1}{{\omega }^{2}} \left( {\omega }^{2}\frac{\partial }{\partial {\omega }}{{\mathcal {P}}}({\omega },{\xi })\right) +{\omega }\frac{\partial {\mathcal {R}}({\omega },{\xi })}{\partial {\omega }}-2{\omega }{\xi }-6-{\xi }\Bigg ]=0,\\ {\mathcal {R}}({\omega },{s})&+\frac{1}{s^{\zeta }}\mathcal {L}_{\xi }\Bigg [-\frac{1}{{\omega }^{2}} \left( {\omega }^{2}\frac{\partial }{\partial {\omega }}{\mathcal {R}}({\omega },{\xi })\right) +{\omega }\frac{\partial ^{2} {{\mathcal {P}}}({\omega },{\xi })}{\partial {\xi }\partial {\omega }}-3{\omega }^{2}+6{\xi }\Bigg ]=0. \end{aligned}$$

The $jth$-truncated term series are

39 $$\begin{aligned} {{\mathcal {P}}}_{{k}}({\omega },s)=&\frac{{\omega }^{2}}{s}+\frac{{\omega }^{2}}{{s}^{2}} +\sum _{i=1}^{{j}}\frac{f_{i}({\omega })}{s^{{i}{\zeta }+2}},\\ {\mathcal {R}}_{{k}}({\omega },s)=&\sum _{i=1}^{{j}}\frac{g_{i}({\omega })}{s^{i{\zeta }+1}}. \end{aligned}$$

And the ${j}th$-LRFs as:

40 $$\begin{aligned} \mathcal {L}_{\xi }Res_{{{\mathcal {P}}},{j}}=&{{\mathcal {P}}}_{j}({\omega },{s}) -\frac{{\omega }^{2}}{s}-\frac{{\omega }^{2}}{{s}^{2}}+\frac{1}{s^{\zeta +1}}\mathcal {L}_{\xi }\Bigg [-\frac{1}{{\omega }^{2}} \frac{\partial }{\partial {\omega }}\left( {\omega }^{2}\frac{\partial }{\partial {\omega }} \mathcal {L}_{s}^{-1}\left( {{\mathcal {P}}}_{j}({\omega },{s})\right) \right) +{\omega }\frac{\partial }{\partial {\omega }}\mathcal {L}_{s}^{-1}\left( {\mathcal {R}}_{j}({\omega },{s})\right) \\&-2{\omega }{\xi }-6-{\xi }\Bigg ],\\ \mathcal {L}_{\xi }Res_{{\mathcal {R}},{j}}=&{\mathcal {R}}_{j}({\omega },{s})+\frac{1}{s^{\zeta }} \mathcal {L}_{\xi }\Bigg [-\frac{1}{{\omega }^{2}}\frac{\partial }{\partial \omega }\left( {\omega }^{2}\frac{\partial }{\partial {\omega }} \mathcal {L}_{s}^{-1}\left( {\mathcal {R}}_{j}({\omega },{s})\right) \right) +{\omega }\frac{\partial ^{2}}{\partial {\xi }\partial {\omega }} \mathcal {L}_{s}^{-1}\left( {{\mathcal {P}}}_{j}({\omega },{s})\right) -3{\omega }^{2}+6{\xi }\Bigg ]. \end{aligned}$$

Putting the ${j}th$ truncated term series of Eq. (39) into the ${j}th$ truncated Laplace residual function of Eq. (40), multiplying the resulting expression by $s^{j{\zeta }+1}$ and then solve the systems $\lim _{{j}\rightarrow {\infty }}s^{j{\zeta }+1}\mathcal {L}Res_{{{\mathcal {P}}},{j}}({\omega },{s})=0$ and $\lim _{{j}\rightarrow {\infty }}s^{j{\zeta }+1}\mathcal {L}Res_{{\mathcal {R}},{j}}({\omega },{s})=0$ to find the unknown coefficients ${\kappa }_{j}({\omega })$ and ${\varrho }_{j}({\omega })$ for ${j}=1,2,3,\ldots$, the following are the first few terms of the approximate solutions

41 $$\begin{aligned} {\kappa }_{1}({\omega })=&0,\\ {\varrho }_{1}({\omega })=&{\omega }^{2},\\ {\kappa }_{2}({\omega })=&0,\\ {\varrho }_{2}({\omega })=&0,\\&\vdots , \end{aligned}$$

Substituting ${\kappa }_{j}({\omega })$ and ${\varrho }_{j}({\omega })$ for ${j}=1,2,3,\ldots$ in Eq. (39), we have

42 $$\begin{aligned} {{\mathcal {P}}}({\omega },s)=&\frac{{\omega }^{2}}{s}+\frac{{\omega }^{2}}{s^{2}} +\frac{0}{s^{{\zeta }+2}}+\frac{0}{s^{2{\zeta }+2}}+\cdots ,\\ {\mathcal {R}}({\omega },s)=&\frac{\omega ^{2}}{s^{{\zeta }+1}}+\frac{0}{s^{{\zeta }+2}}+\cdots . \end{aligned}$$

Utilizing inverse LT on Eq. (42), we get the approximate solution as

43 $$\begin{aligned} {{\mathcal {P}}}({\omega },{\xi })=&{\omega }^{2}+{\omega }^{2}{\xi },\\ {\mathcal {R}}({\omega },{\xi })=&\frac{\omega ^{2}{\xi }^{\zeta }}{\Gamma ({\zeta }+1)}. \end{aligned}$$

Putting $\zeta =1$, we get the exact solution which is given in Eq. (37).

## Results and disscusions

**Figure 9:**
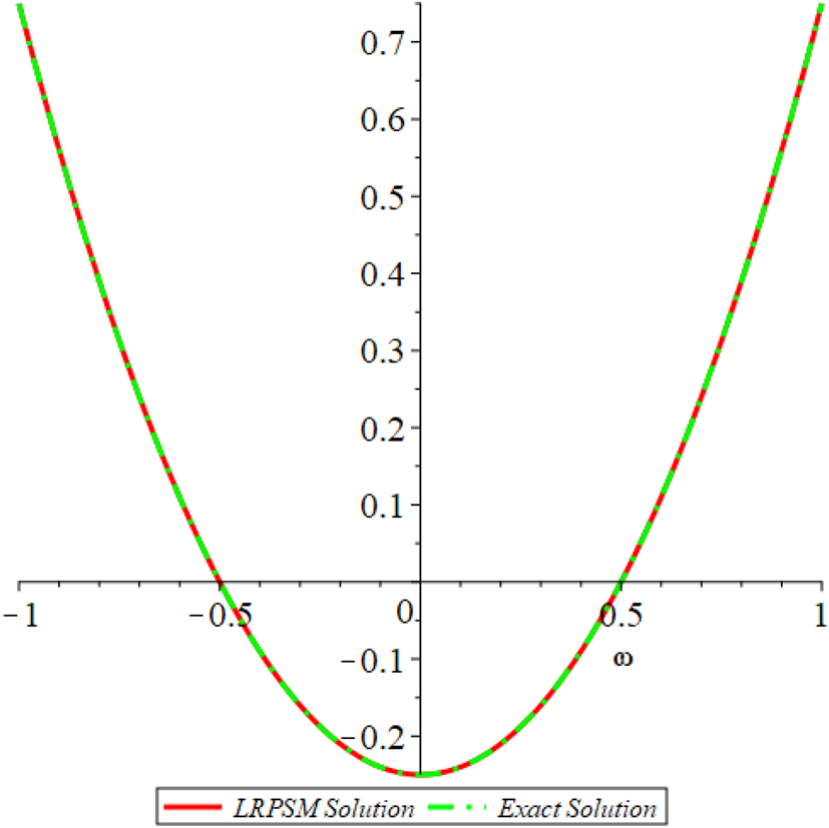
2D plots of ${{\mathcal {P}}}({\omega },{\xi })$ LRPSM and exact solutions for $\zeta =1$ for problem 4.2.

**Figure 10:**
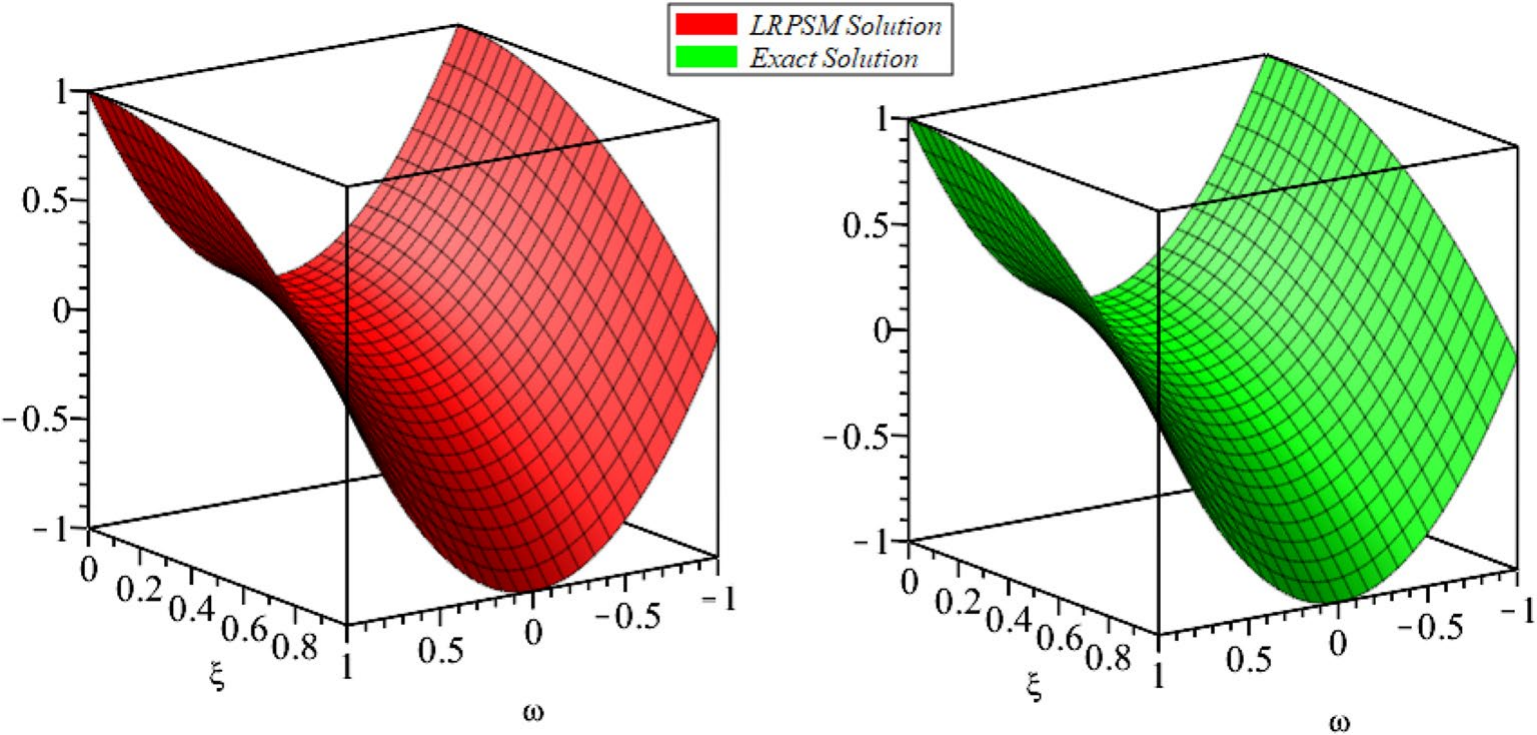
3D surfaces of ${{\mathcal {P}}}({\omega },{\xi })$ LRPSM and exact solutions for $\zeta =1$ for problem 4.2.

**Figure 11:**
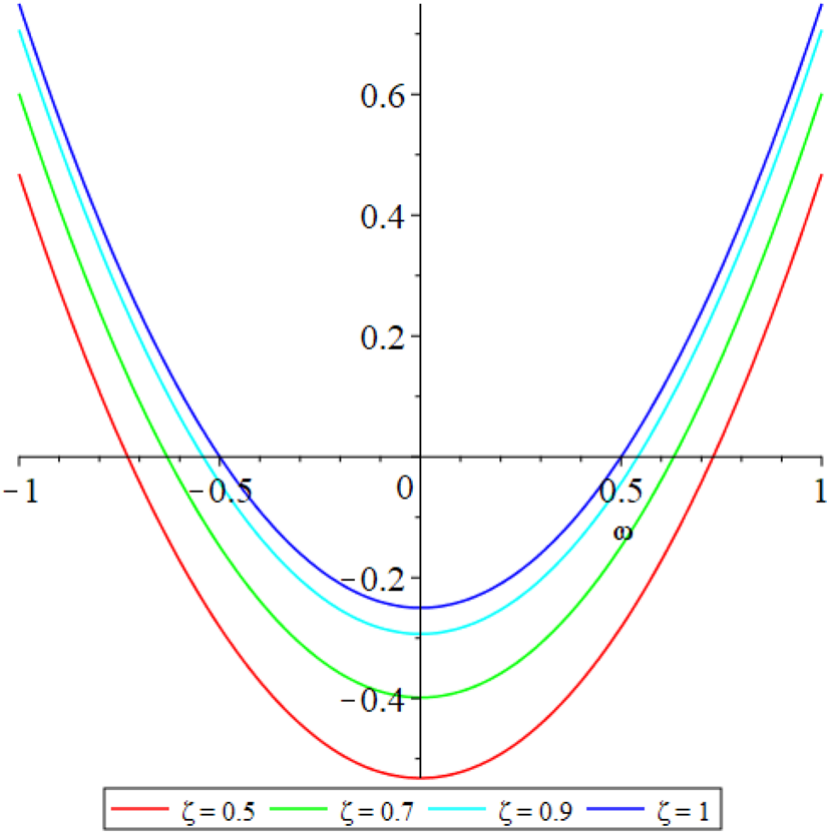
2D plots of ${{\mathcal {P}}}({\omega },{\xi })$ LRPSM solution with different values of $\zeta$ for problem 4.2.

**Figure 12:**
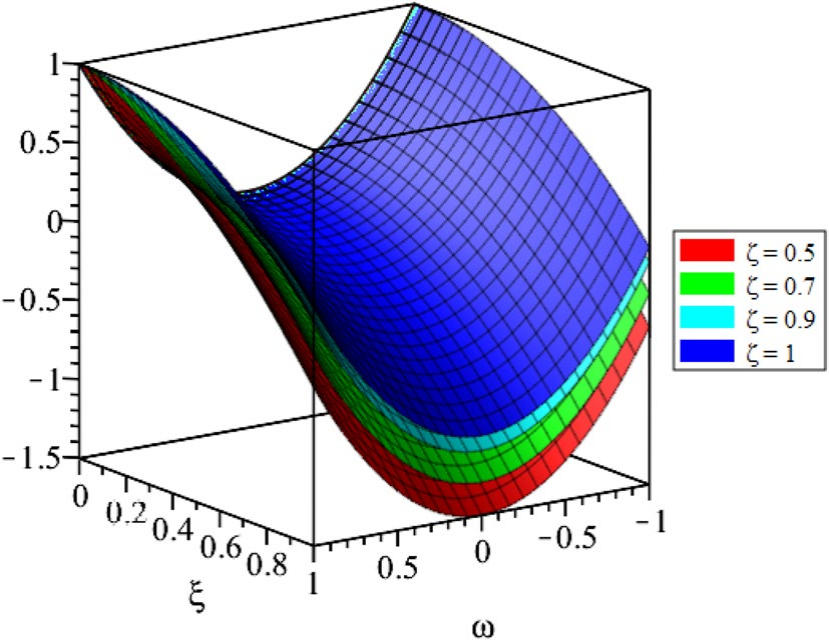
3D surfaces of ${{\mathcal {P}}}({\omega },{\xi })$ LRPSM solution with different values of $\zeta$ for problem 4.2.

**Figure 13:**
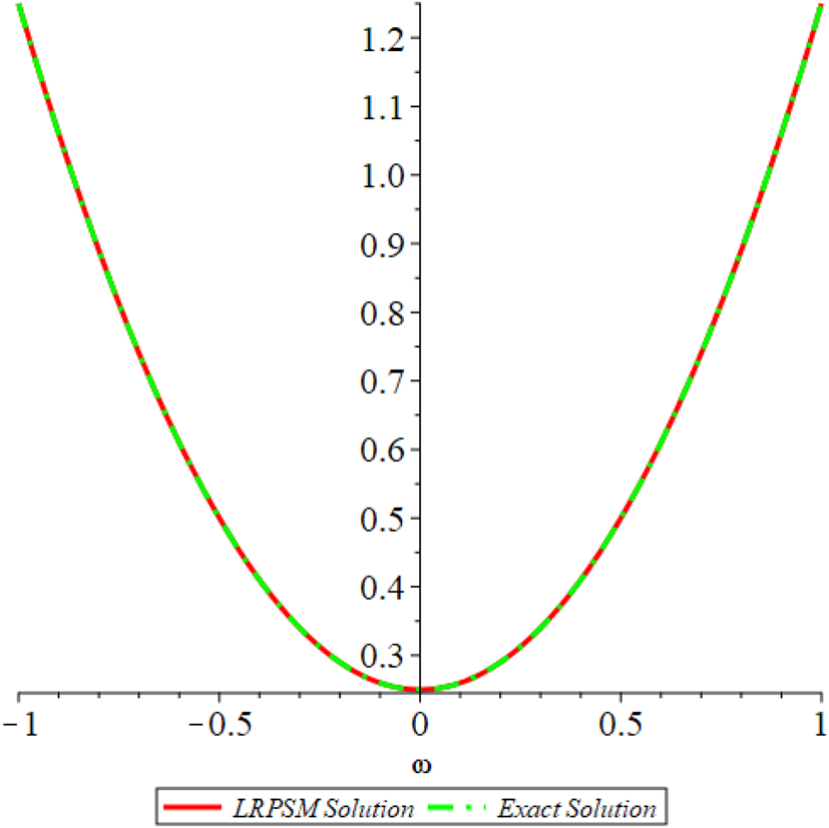
2D plots of ${{\mathcal {R}}}({\omega },{\xi })$ LRPSM and exact solutions for $\zeta =1$ for problem 4.2.

**Figure 14:**
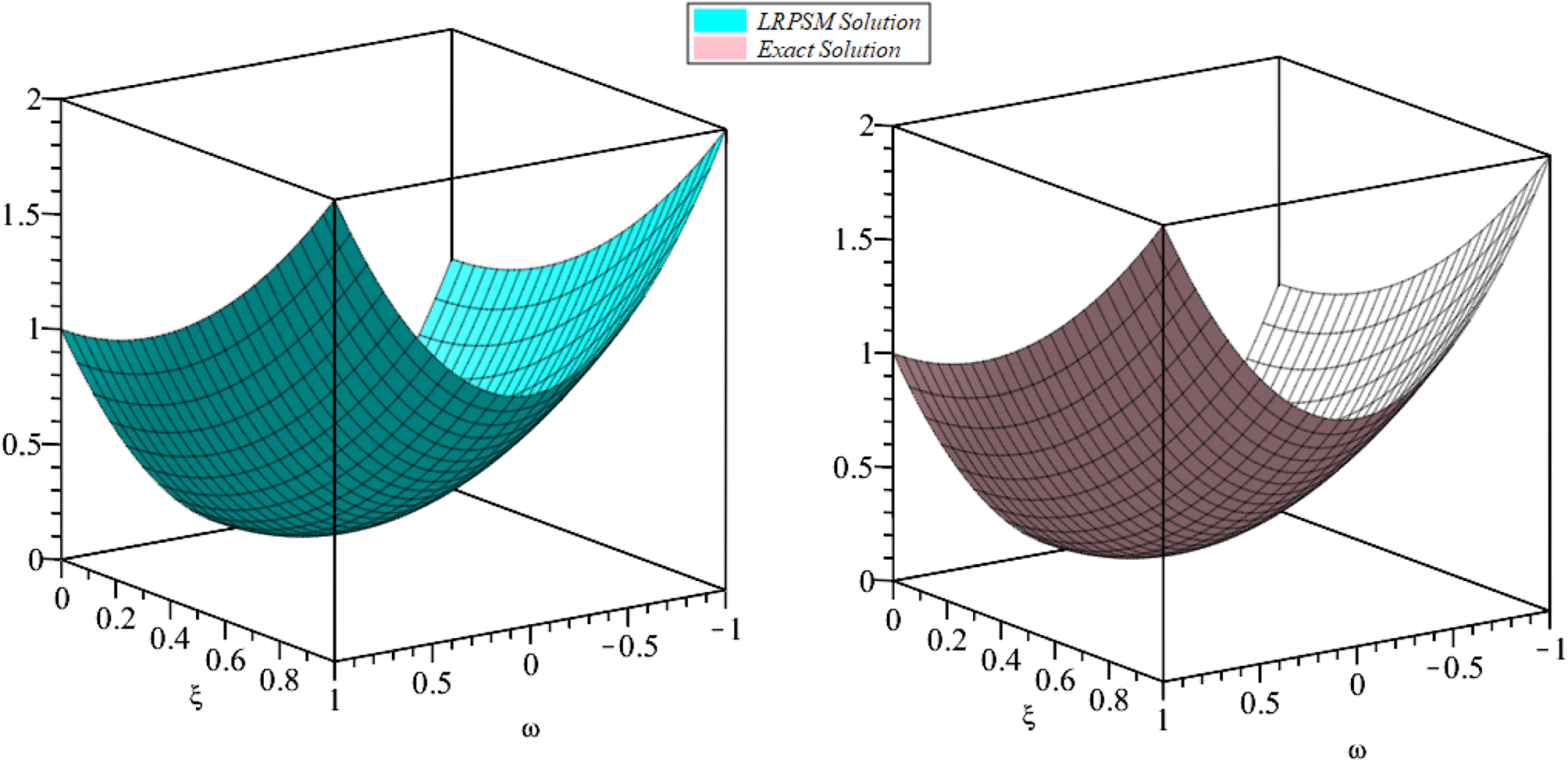
3D surfaces of ${{\mathcal {R}}}({\omega },{\xi })$ LRPSM and exact solutions for $\zeta =1$ for problem 4.2.

**Figure 15:**
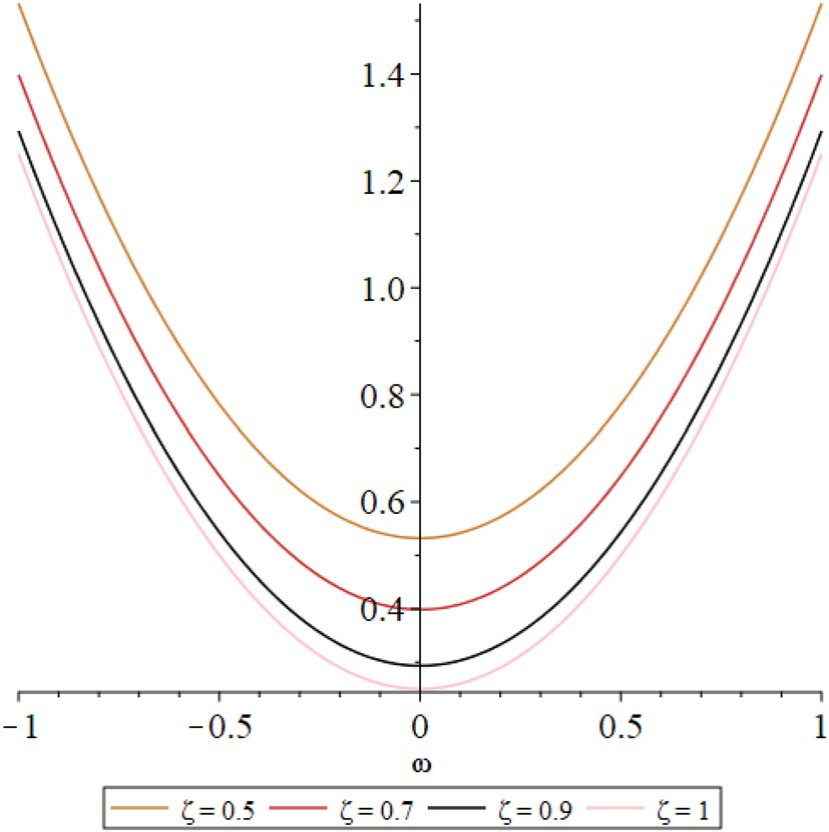
2D plots of ${{\mathcal {R}}}({\omega },{\xi })$ LRPSM solution with different values of $\zeta$ for problem 4.2.

**Figure 16:**
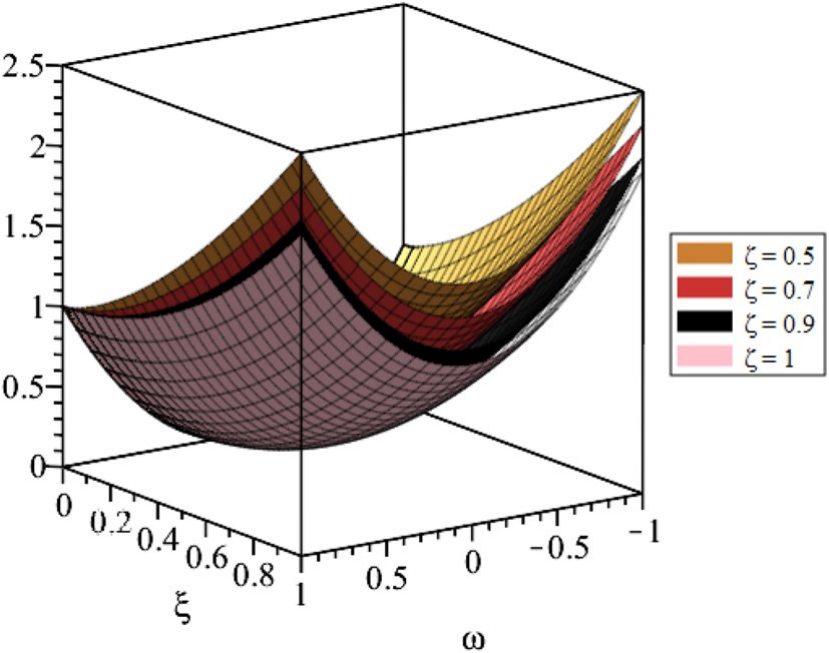
3D surfaces of ${{\mathcal {R}}}({\omega },{\xi })$ LRPSM solution with different values of $\zeta$ for problem 4.2.

**Figure 17:**
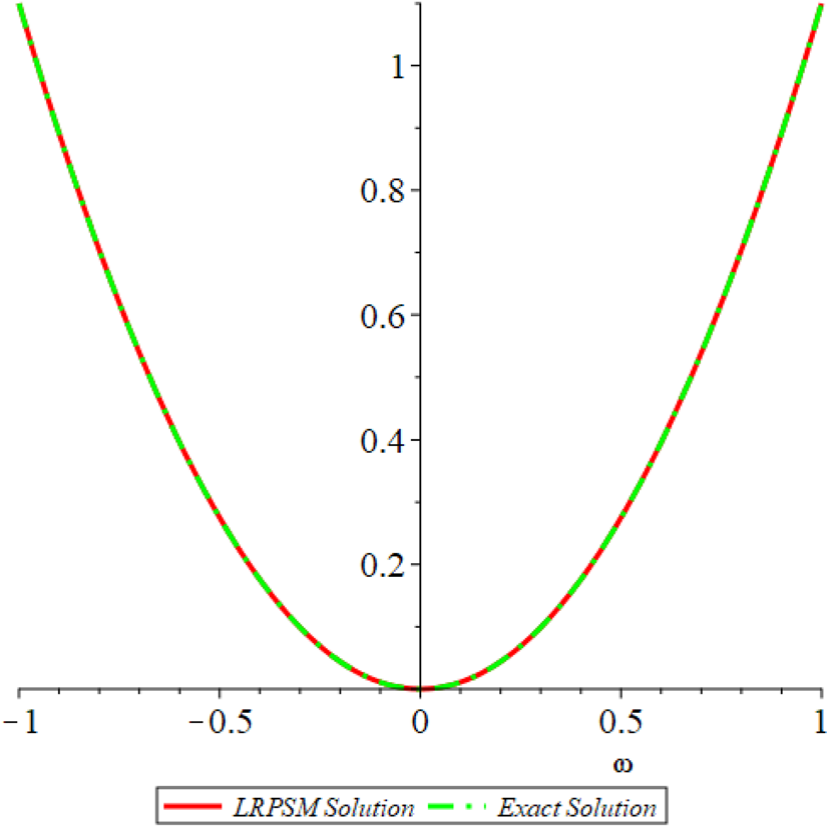
2D plots of ${{\mathcal {P}}}({\omega },{\xi })$ LRPSM and exact solutions for $\zeta =1$ for problem 4.3.

**Figure 18:**
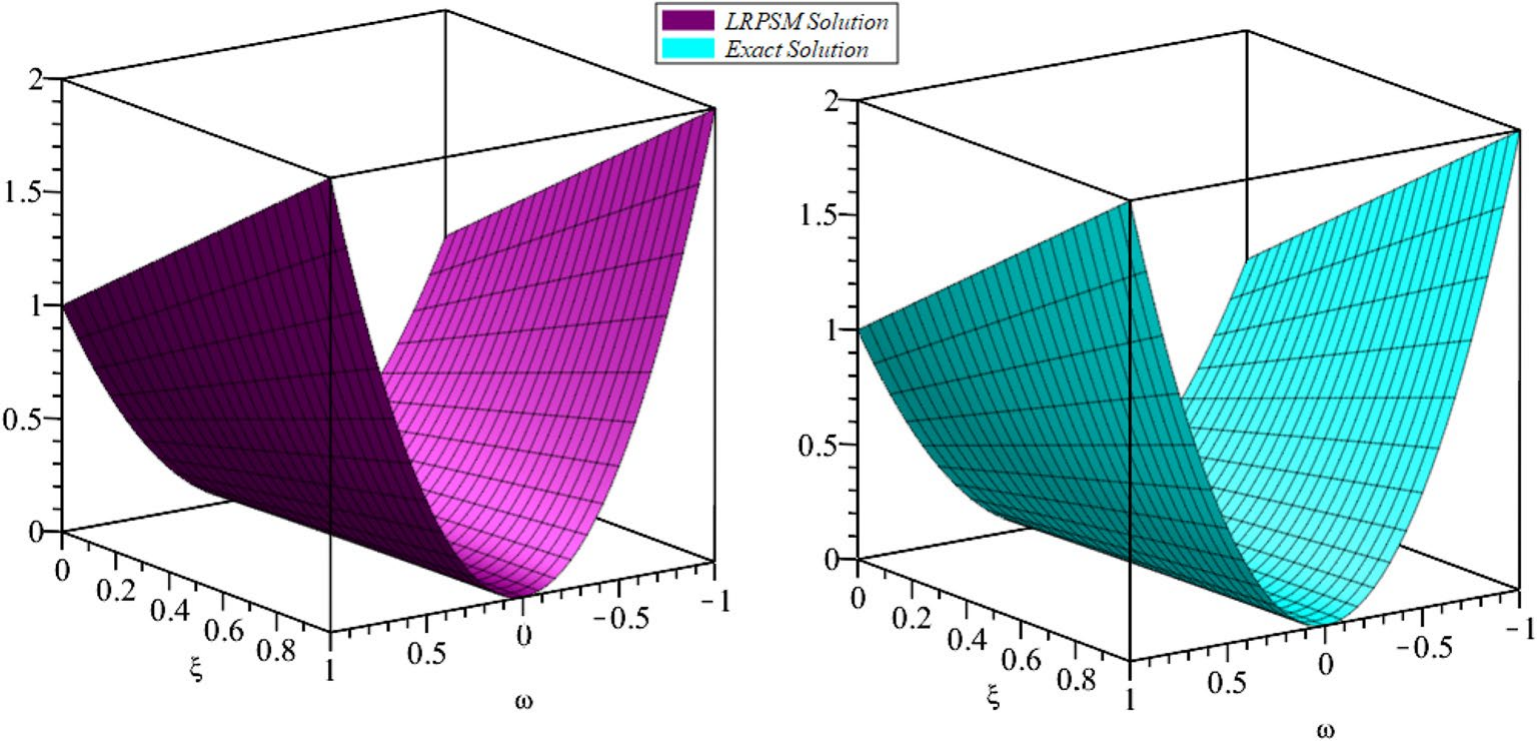
3D surfaces of ${{\mathcal {P}}}({\omega },{\xi })$ LRPSM and exact solutions for $\zeta =1$ for problem 4.3.

**Figure 19:**
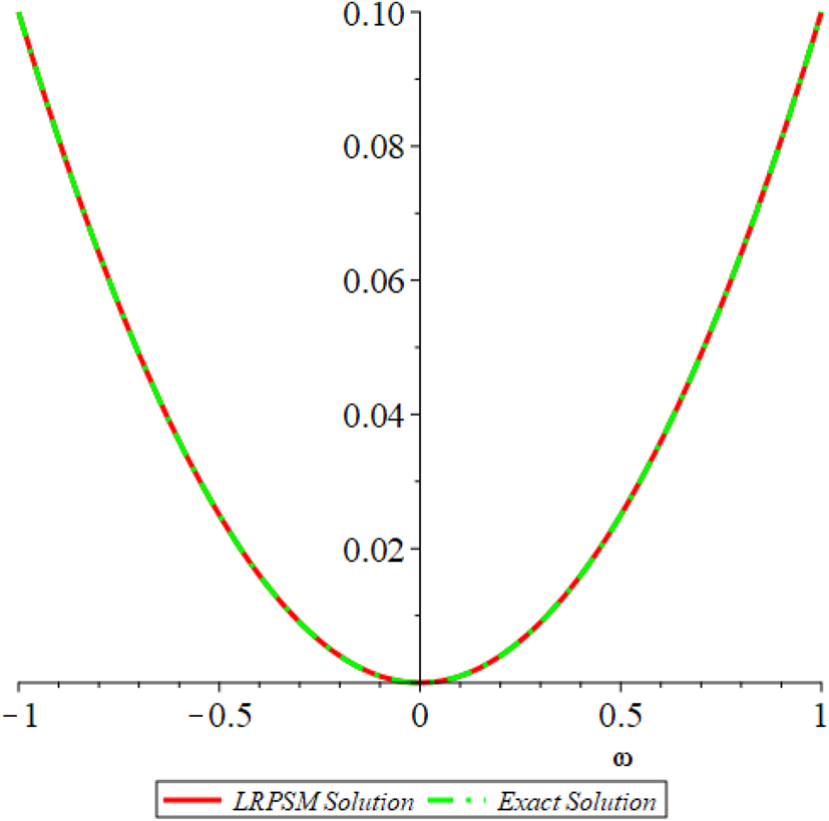
2D plots of ${{\mathcal {R}}}({\omega },{\xi })$ LRPSM and exact solutions for $\zeta =1$ for problem 4.3.

**Figure 20:**
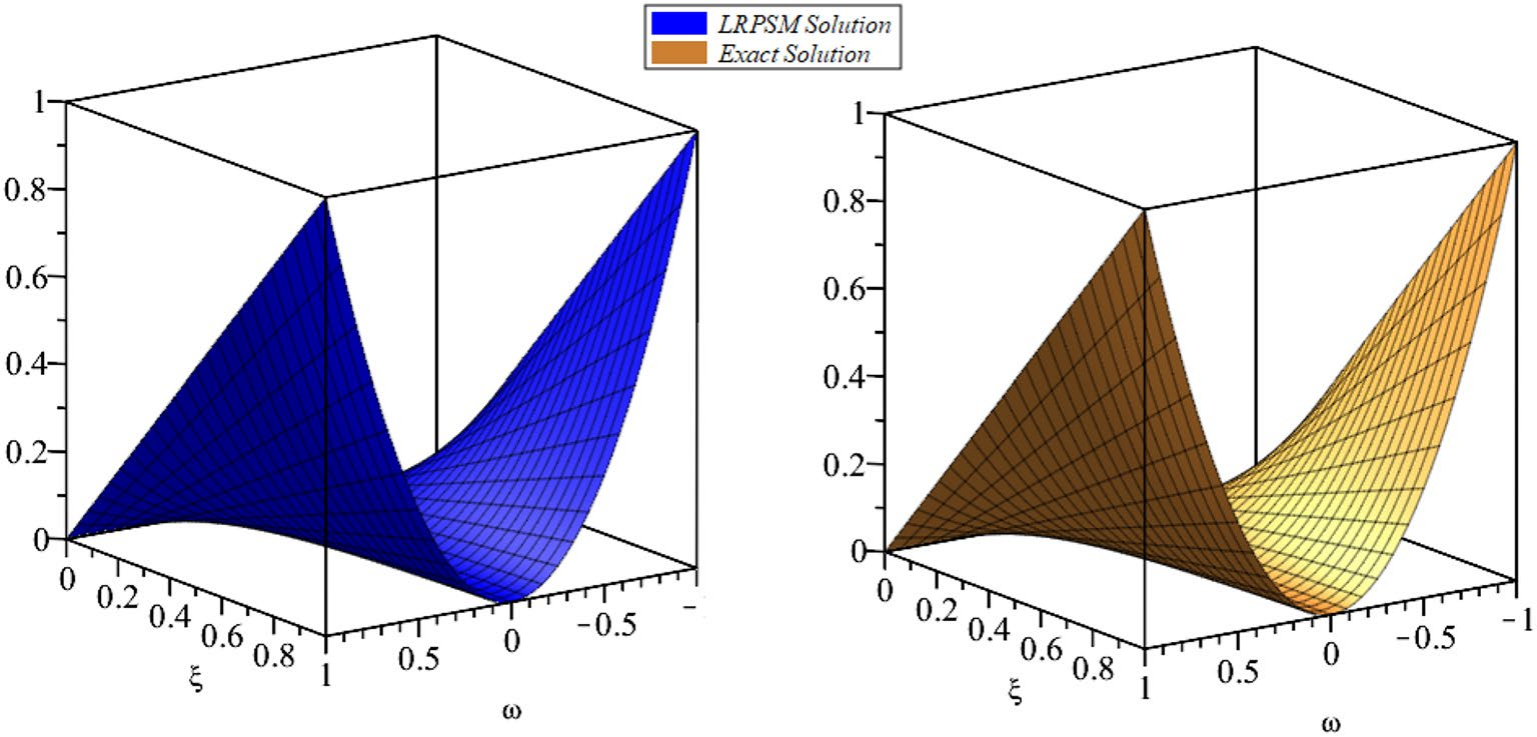
3D surfaces of ${{\mathcal {R}}}({\omega },{\xi })$ LRPSM and exact solutions for $\zeta =1$ for problem 4.3.

**Figure 21:**
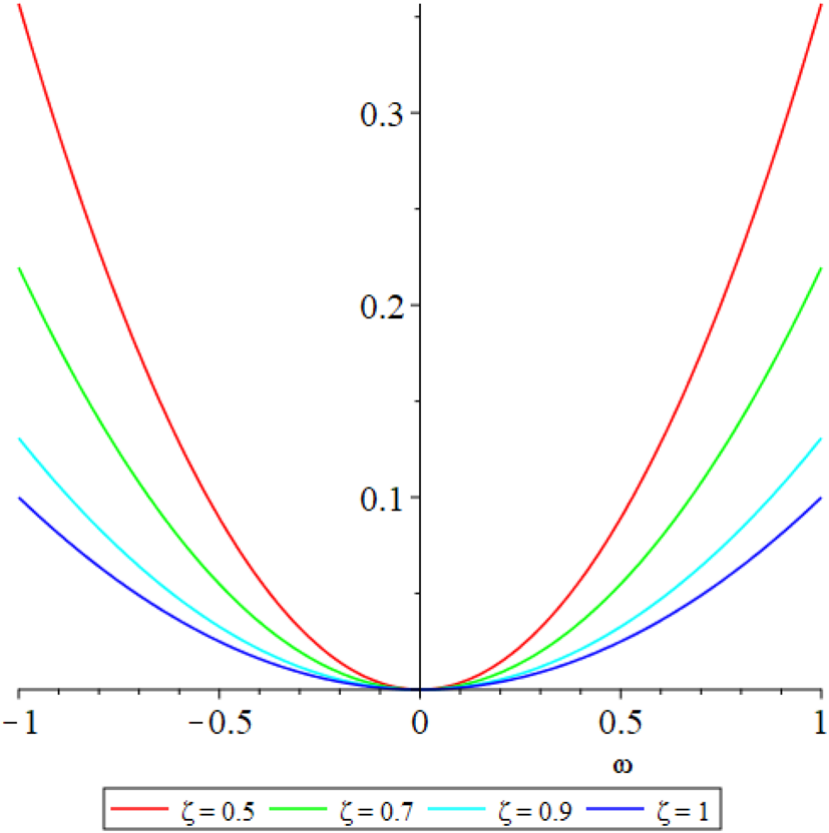
2D plots of ${{\mathcal {R}}}({\omega },{\xi })$ LRPSM solution with different values of $\zeta$ for problem 4.3.

**Figure 22:**
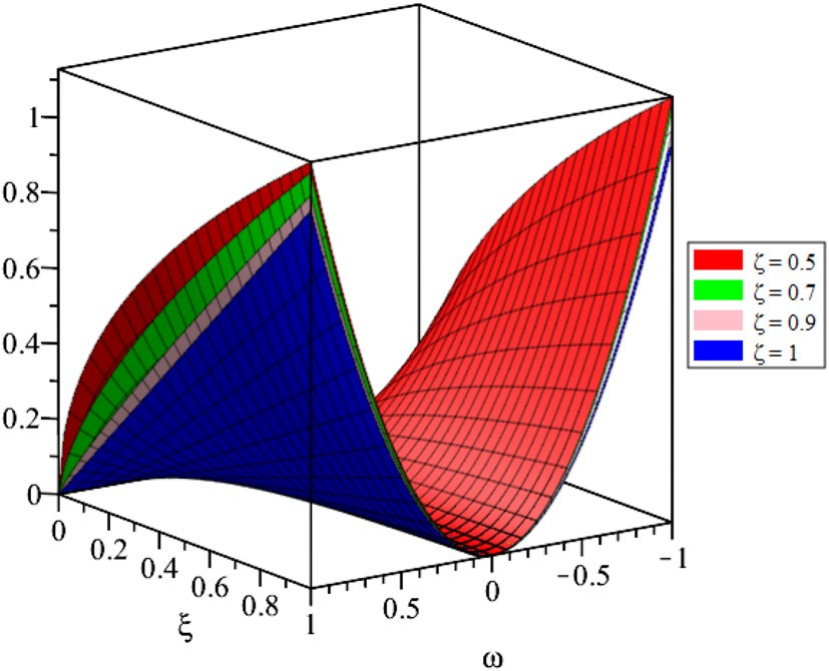
3D surfaces of ${{\mathcal {R}}}({\omega },{\xi })$ LRPSM solution with different values of $\zeta$ for problem 4.3.

In this section, the numerical solutions of the coupled system considered in Problem 4.1 are discussed, which is given in Tables 1 and 2. Further, in Tables 1 and 2. The effectiveness of the technique is indicated, as the number of iterations increases, the solution approaches to the exact solution. We can see from the tables, the present technique has the higher accuracy. The 2D and 3D plots are presented to highlighted the LRPSM results at different values of parameters. The 2D plots of problem 4.1 at various fractional-order for $\mathcal {P}(\omega ,\xi )$ is shown in Fig. 1 and for $\mathcal {R}(\omega ,\xi )$ is shown in Fig. 5 and the solution in 3D surfaces are shown in Figs. 3 and 7. The comparison of LRPSM and exact solutions are plotted in Figs. 2, 4, 6 and 8 as 2D and 3D respectively. Similarly, the 2D and 3D plots of Problems 4.2 and 4.3 at various fractional-order are discussed in Figs. 9, 10, 11, 12, 13, 14 and 15, respectively. Meanwhile, the response of the LRPSM solution in term of AE for various arbitrary orders are shown in Tables 3, 4, 5 and 6 respectively for Problem 4.2 and Problem 4.3. Also for Problem 4.3, the solutions are plotted in 2D and 3D graphs in Figs. 16, 17, 18, 19, 20, 21, and 22. With the help of FC, we can study and analyze the physical behavior of non-linear problem by simulating and displaying its physical properties. The suggested technique is more suitable and efficient in analyzing complex coupled fractional-order problems. All the numerical calculations are done by Maple 2020.

**Table 1:** Numerical simualtion for ${{\mathcal {P}}}({\omega },{\xi })$ at $\zeta =1$ of Example 4.1.

ine ${\xi }$	${\omega }$	$|{{\mathcal {P}}}_{exact}-{{\mathcal {P}}}^{(4)}|$	$|{{\mathcal {P}}}_{exact} -{{\mathcal {P}}}^{(5)}|$	$|{{\mathcal {P}}}_{exact}-{{\mathcal {P}}}^{(6)}|$
ine	0.2	1.00 $\times \; 10^{-7}$	2.0 $\times \; 10^{-9}$	0
	0.4	1.22 $\times \; 10^{-7}$	2.0 $\times \; 10^{-9}$	0
0.1	0.6	1.49 $\times \; 10^{-7}$	3.0 $\times \; 10^{-9}$	0
	0.8	1.83 $\times\; 10^{-7}$	2.0 $\times \; 10^{-9}$	1.0 $\times \; 10^{-9}$
	1	2.22 $\times \; 10^{-7}$	5.0 $\times \; 10^{-9}$	1.0 $\times \; 10^{-9}$
ine	0.2	9.5399 $\times \; 10^{-6}$	3.999 $\times\; 10^{-7}$	1.43 $\times \; 10^{-8}$
	0.4	1.1653 $\times \; 10^{-5}$	4.88 $\times \; 10^{-7}$	1.8 $\times \; 10^{-8}$
0.25	0.6	1.4231 $\times \; 10^{-5}$	5.97 $\times \; 10^{-7}$	2.10 $\times \; 10^{-8}$
	0.8	1.7382 $\times \;10^{-5}$	7.29 $\times\; 10^{-7}$	2.6$\times 10^{-8}$
	1	2.1231 $\times \; 10^{-5}$	8.90 $\times \; 10^{-7}$	3.2 $\times \; 10^{-8}$

**Table 2:** Numerical simualtion for ${\mathcal {R}}({\omega },{\xi })$ at $\beta =1$ of Example 4.1.

ine ${\xi }$	${\omega }$	$|{\mathcal {R}}_{exact}-{\mathcal {R}}^{(4)}|$	$|{\mathcal {R}}_{exact}-{\mathcal {R}}^{(5)}|$	$|{\mathcal {R}}_{exact}-{\mathcal {R}}^{(6)}|$
ine	0.2	3.4807 $\times \; 10^{-6}$	6.93 $\times \;10^{-8}$	1.1 $\times \;10^{-9}$
	0.4	2.8499 $\times \; 10^{-6}$	5.69 $\times \; 10^{-8}$	1.0 $\times \; 10^{-9}$
0.1	0.6	2.3332 $\times \; 10^{-6}$	4.65 $\times \; 10^{-8}$	8.0 $\times \; 10^{-9}$
	0.8	1.9103 $\times \; 10^{-6}$	3.81 $\times\; 10^{-8}$	7.0 $\times\; 10^{-9}$
	1	1.5640 $\times \; 10^{-6}$	3.12 $\times \; 10^{-8}$	5.0 $\times \; 10^{-9}$
ine	0.2	1.40208 $\times \; 10^{-4}$	6.951 $\times \; 10^{-6}$	2.88 $\times \; 10^{-7}$
	0.4	1.147924 $\times \;10^{-4}$	5.6908 $\times\; 10^{-6}$	2.357 $\times \;10^{-7}$
0.25	0.6	9.39840 $\times \;10^{-5}$	4.6592 $\times\; 10^{-6}$	1.930 $\times \;10^{-7}$
	0.8	7.69477 $\times \; 10^{-5}$	3.8147 $\times \; 10^{-6}$	1.581 $\times \; 10^{-7}$
	1	6.29993 $\times \; 10^{-5}$	3.1231 $\times \; 10^{-6}$	1.293 $\times \; 10^{-7}$

**Table 3:** AE for ${{\mathcal {P}}}({\omega },{\xi })$ at $\zeta =1$ of Example 4.1.

${\xi }$	${\omega }$	AE at $\zeta =0.5$	AE at $\zeta =0.7$	AE at $\zeta =0.9$	AE at $\zeta =1$
0.1	0	0.03757664309	0.01583389133	0.00377866212	0
	0.1	0.03757664309	0.01583389133	0.00377866212	0
	0.2	0.03757664309	0.01583389133	0.00377866212	0
	0.3	0.0375766431	0.0158338913	0.0037786621	0
	0.4	0.0375766431	0.0158338913	0.0037786621	0
	0.5	0.0375766431	0.0158338913	0.0037786621	0
	0.6	0.0375766431	0.0158338913	0.0037786621	0
	0.7	0.0375766431	0.0158338913	0.0037786621	0
	0.8	0.0375766431	0.0158338913	0.0037786621	0
	0.9	0.0375766431	0.0158338913	0.0037786621	0
	1	0.0375766431	0.0158338913	0.0037786621	0

**Table 4:** AE for ${{\mathcal {R}}}({\omega },{\xi })$ at $\zeta =1$ of Example 4.1.

${\xi }$	${\omega }$	AE at $\zeta =0.5$	AE at $\zeta =0.7$	AE at $\zeta =0.9$	AE at $\zeta =1$
0.1	0	0.03757664309	0.01583389133	0.00377866212	0
	0.1	0.03757664309	0.01583389133	0.00377866212	0
	0.2	0.03757664309	0.01583389133	0.00377866212	0
	0.3	0.0375766431	0.0158338913	0.0037786621	0
	0.4	0.0375766431	0.0158338913	0.0037786621	0
	0.5	0.0375766431	0.0158338913	0.0037786621	0
	0.6	0.0375766431	0.0158338913	0.0037786621	0
	0.7	0.0375766431	0.0158338913	0.0037786621	0
	0.8	0.0375766431	0.0158338913	0.0037786621	0
	0.9	0.0375766431	0.0158338913	0.0037786621	0
	1	0.0375766431	0.0158338913	0.0037786621	0

**Table 5:** AE for ${{\mathcal {R}}}({\omega },{\xi })$ at $\zeta =1$ of Example 4.3.

${\xi }$	${\omega }$	LRPSM Solution at $\zeta =1$	Exact Solution	|LRPSM-Exact|
0.1	0	0	0	0
	0.1	0.011	0.011	0
	0.2	0.044	0.044	0
	0.3	0.099	0.099	0
	0.4	0.176	0.176	0
	0.5	0.275	0.275	0
	0.6	0.396	0.396	0
	0.7	0.539	0.539	0
	0.8	0.704	0.704	0
	0.9	0.891	0.891	0
	1	1.100	1.100	0

**Table 6:** AE for ${{\mathcal {R}}}({\omega },{\xi })$ at $\zeta =1$ of Example 4.3.

${\xi }$	${\omega }$	LRPSM Solution at $\zeta =1$	Exact Solution	|LRPSM-Exact|
0.1	0	0	0	0
	0.1	0.001	0.001	0
	0.2	0.004	0.004	0
	0.3	0.009	0.009	0
	0.4	0.016	0.016	0
	0.5	0.025	0.025	0
	0.6	0.036	0.036	0
	0.7	0.049	0.049	0
	0.8	0.064	0.064	0
	0.9	0.081	0.081	0
	1	0.100	0.100	0

## Conclusion

The current research explores a broader and more practical concept within the fractional analogue of certain thermo-elasticity systems. Initially, the thermo-elasticity systems are expressed in their Caputo fractional definition and then effectively examined for their practical dynamics using a precise approach. The technique directly addresses non-linearity, a rarity in other existing methods. Employing Laplace transformation to simplify the given problems, the residual power series method is then utilized to attain the complete solution. The results demonstrate full compatibility with the actual dynamics of the suggested problems. Fractional solutions, in particular, offer numerous options for describing the practical dynamics of problems. This study can significantly contribute to the analysis of other highly non-linear complex phenomena.

## References

[1] Oldham, K. & Spanier, J. *The Fractional Calculus: Theory and Applications of Differentiation and Integration to Arbitrary Order* (Academic Press, 1974).

[2] Miller, K. S. & Ross, B. *An Introduction to Fractional Calculus and Fractional Differential Equations* (Wiley, 1993).

[3] Podlubny, I. *Fractional Differential Equations* (Academic Press, 1999).

[4] Kilbas, A. A., Srivastava, H. M. & Trujillo, J. J. *Theory and Applications of Fractional Differential Equations* (Elsevier, 2006).

[5] Baleanu, D. *et al.* (eds) *New Trends in Nanotechnology and Fractional Calculus Applications* (Springer, 2010).

[6] Mainardi, F. *Fractional Calculus and Waves in Linear Viscoelasticity* (Imperial College Press, 2010).

[7] Almeida, R., Tavares, D. & Torres, D. F. *The Variable-Order Fractional Calculus of Variations* (Springer International Publishing, 2019).

[8] Kilbasi, A. A. & Saigo, M. On Mittag-Leffler type function, fractional calculas operators and solutions of integral equations. *Integr. Transforms Special Funct.* **4**(4), 355–370 (1996).

[9] Esen, A., Sulaiman, T. A., Bulut, H. & Baskonus, H. M. Optical solitons and other solutions to the conformable space-time fractional Fokas-Lenells equation. *Optik* **167**, 150–156 (2018).

[10] Veeresha, P., Prakasha, D. G. & Baskonus, H. M. Solving smoking epidemic model of fractional order using a modified homotopy analysis transform method. *Math. Sci.* **13**(2), 115–128 (2019).

[11] Baleanu, D., Wu, G. C. & Zeng, S. D. Chaos analysis and asymptotic stability of generalized Caputo fractional differential equations. *Chaos Solitons Fract.* **102**, 99–105 (2017).

[12] Farayola, M. F., Shafie, S., Siam, F. M. & Khan, I. Mathematical modeling of radiotherapy cancer treatment using Caputo fractional derivative. *Comput. Methods Programs Biomedicine* **188**, 105306 (2020). 10.1016/j.cmpb.2019.105306 31901851

[13] Baskonus, H. M., Sulaiman, T. A. & Bulut, H. On the new wave behavior to the Klein-Gordon-Zakharov equations in plasma physics. *Indian J. Phys.* **93**(3), 393–399 (2019).

[14] Shah, S. A. A., Khan, M. A., Farooq, M., Ullah, S. & Alzahrani, E. O. A fractional order model for Hepatitis B virus with treatment via Atangana-Baleanu derivative. *Phys. A Stat. Mech. Appl.* **538**, 122636 (2020).

[15] Khater, M. M. A., Raghda, A. M. A. & Lu, D. Computational and numerical simulations for the nonlinear fractional Kolmogorov-Petrovskii-Piskunov (FKPP) equation. *Physica Scripta* **95**(5), 055213 (2020).

[16] Singh, B. K. & Kumar, P. Fractional variational iteration method for solving fractional partial differential equations with proportional delay. *Int. J. Differ. Equ.* **2017** (2017).

[17] Sadighi, A. & Ganji, D. D. A study on one dimensional nonlinear thermoelasticity by Adomian decomposition method. *World J. Model. Simul.* **4**(1), 19–25 (2008).

[18] Gao, W., Veeresha, P., Prakasha, D. G., Senel, B. & Baskonus, H. M. Iterative method applied to the fractional nonlinear systems arising in thermoelasticity with Mittag-Leffler kernel. *Fractals* **28**(08), 2040040 (2020).

[19] Li, X. & Li, S. A fast element-free Galerkin method for the fractional diffusion-wave equation. *Appl. Math. Lett.* **122**, 107529 (2021).

[20] Saha Ray, S. A new approach by two-dimensional wavelets operational matrix method for solving variable-order fractional partial integro-differential equations. *Numer. Methods Partial Differ. Equ.* **37**(1), 341–359 (2021).

[21] Sarwe, D. U. & Kulkarni, V. S. Analysis of nonlinear systems arise in thermoelasticity using fractional natural decomposition scheme. *Math. Methods Appl. Sci.* **45**(1), 341–358 (2022).

[22] Candoğan, K., Altuntas, E. G. & İğci, N. Authentication and quality assessment of meat products by Fourier-transform infrared (FTIR) spectroscopy. *Food Eng. Rev.* **13**(1), 66–91 (2021).

[23] Momani, S. & Odibat, Z. Comparison between the homotopy perturbation method and the variational iteration method for linear fractional partial differential equations. *Comput. Math. Appl.* **54**(7–8), 910–919 (2007).

[24] Fahad, H. M. & Fernandez, A. Operational calculus for Caputo fractional calculus with respect to functions and the associated fractional differential equations. *Appl. Math. Comput.* **409**, 126400 (2021).

[25] Ahmed, S. A., Elzaki, T. M., Elbadri, M. & Mohamed, M. Z. Solution of partial differential equations by new double integral transform (Laplace-Sumudu transform). *Ain Shams Eng. J.* **12**(4), 4045–4049 (2021).

[26] Aljahdaly, N. H. & El-Tantawy, S. A. On the multistage differential transformation method for analyzing damping Duffing oscillator and its applications to plasma physics. *Mathematics* **9**(4), 432 (2021).

[27] Momani, S., Djeddi, N., Al-Smadi, M. & Al-Omari, S. Numerical investigation for Caputo-Fabrizio fractional Riccati and Bernoulli equations using iterative reproducing kernel method. *Appl. Numer. Math.* **170**, 418–434 (2021).

[28] El-Ajou, A. Adapting the Laplace transform to create solitary solutions for the nonlinear time-fractional dispersive PDEs via a new approach. *Eur. Phys. J. Plus* **136**(2), 1–22 (2021).

[29] Eriqat, T., El-Ajou, A., Moa’ath, N. O., Al-Zhour, Z. & Momani, S. A new attractive analytic approach for solutions of linear and nonlinear neutral fractional pantograph equations. *Chaos Solitons Fract.* **138**, 109957 (2020).

[30] Burqan, A., El-Ajou, A., Saadeh, R. & Al-Smadi, M. A new efficient technique using Laplace transforms and smooth expansions to construct a series solution to the time-fractional Navier-Stokes equations. *Alex. Eng. J.* **61**(2), 1069–1077 (2022).

[31] El-Ajou, A., Al-Smadi, M., Oqielat, M., Momani, S. & Hadid, S. Smooth expansion to solve high-order linear conformable fractional PDEs via residual power series method: Applications to physical and engineering equations. *Ain Shams Eng. J.* (2020) (**in Press**).

[32] El-Ajou, A., Oqielat, M., Al-Zhour, Z. & Momani, S. A class of linear non-homogenous higher order matrix fractional differential equations: analytical solutions and new technique. *Fract. Calc. Appl. Anal.* **23**(2), 356–377 (2020).

[33] Shqair, M., El-Ajou, A. & Nairat, M. Analytical solution for multi-energy groups of neutron diffusion equations by a residual power series method. *Mathematics* **7**(7), 633 (2019).

[34] El-Ajou, A., Al-Zhour, Z., Oqielat, M., Momani, S. & Hayat, T. Series solutions of non- linear conformable fractional KdV-Burgers equation with some applications. *Eur. Phys. J. Plus* **134**(8), 402 (2019).

[35] Oqielat, M., El-Ajou, A., Al-Zhour, Z., Alkhasawneh, R. & Alrabaiah, H. Series solutions for nonlinear time-fractional Schrödinger equations: Comparisons between conformable and Caputo derivatives. *Alex. Eng. J.*10.1016/j.aej.2020.01.023 (2020).

[36] El-Ajou, A., Oqielat, M., Al-Zhour, Z. & Momani, S. Analytical numerical solutions of the fractional multi-pantograph system: Two attractive methods and comparisons. *Results Phys.* **14**(1), 102500 (2019).

[37] Irwaq, I. A., Alquran, M., Ali, M., Jaradat, I. & Noorani, M. S. M. Attractive new fractional-integer power series method for solving singularly perturbed differential equations involving mixed fractional and integer derivatives. *Results Phys.* **20**, 103780 (2021).

[38] Alquran, M., Ali, M., Alsukhour, M. & Jaradat, I. Promoted residual power series technique with Laplace transform to solve some time-fractional problems arising in physics. *Results Phys.* **19**, 103667 (2020).

[39] Caputo, M. Linear models of dissipation whose Q is almost frequency independent-II. *Geophys. J. Int.* **13**, 529–539 (1967).

[40] Hanna, J. & Rowland, J. *Fourier Series, Transforms, and Boundary Value Problems* (Wiley, 1990).

[41] Arqub, O. A., El-Ajou, A. & Momani, S. Construct and predicts solitary pattern solutions for nonlinear time-fractional dispersive partial differential equations. *J. Comput. Phys.* **293**, 385–399 (2015).

[42] Gadain, H. E. Coupled singular and non singular thermoelastic system and Double Laplace Decomposition method. *New Trends Math Sci.* **4**(3), 212–222 (2016).

